# From No-Show Prediction to Operational Action: A Retrospective Secondary Analysis of Calibrated, Duration-Weighted Dental Appointment Prioritization

**DOI:** 10.3390/healthcare14213731

**Published:** 2026-11-03

**Authors:** Ștefan Lucian Burlea, Călin Gheorghe Buzea, Florin Nedeff, Diana Mirilă, Valentin Nedeff, Maricel Agop, Irina Nica, Giani Gabriel Condurache

**Affiliations:** 1 Dentoalveolar Surgery, Faculty of Medicine, University of Medicine and Pharmacy “Grigore T. Popa” Iași, 700115 Iași, Romania; stefan.burlea@umfiasi.ro; 2 National Institute of Research and Development for Technical Physics—IFT Iași, 700050 Iași, Romania; calinb2003@yahoo.com; 3 Clinical Emergency Hospital “Prof. Dr. Nicolae Oblu” Iași, 700309 Iași, Romania; 4 Department of Environmental Engineering, Mechanical Engineering and Agritourism, Faculty of Engineering, “Vasile Alecsandri” University of Bacău, 600115 Bacău, Romania; florin_nedeff@ub.ro (F.N.); m.agop@yahoo.com (M.A.); 5 Department Odontology-Periodontology, Fixed Prosthesis Faculty of Dental Medicine, University of Medicine and Pharmacy “Grigore T. Popa” Iași, 700115 Iași, Romania; 6 Faculty of Medicine and Pharmacy, “Dunarea de Jos” University of Galati, 47 Domnească Street, 800008 Galati, Romania; geany_rbc@yahoo.com

**Keywords:** dental appointment management, patient no-show, operational efficiency, predictive modeling, probability calibration, scheduled capacity, healthcare management

## Abstract

**Highlights:**

**What are the main findings?**

- Combining calibrated no-show probability with scheduled appointment duration identified a larger proportion of scheduled appointment minutes associated with subsequent non-attendance compared with prioritization based on probability or duration alone. At a 10% intervention budget, the duration-weighted strategy captured 23.04% of scheduled no-show minutes versus 15.88% for probability-only targeting and 18.49% for duration-only targeting.
- The operational advantage of duration-weighted prioritization was consistent across intervention budgets from 5% to 30% and remained evident under rolling-origin temporal validation, supporting the robustness of the resource-oriented ranking approach.

**What are the implications of the main findings?**

- Dental appointment management may benefit from shifting the focus from predicting individual no-shows alone to prioritizing appointments according to the amount of scheduled appointment duration exposed to non-attendance, particularly when administrative intervention resources are limited.
- Calibrated, duration-weighted risk scores could support more efficient targeting of confirmation, reminder, patient-contact, or appointment-recovery actions; however, prospective studies are still needed to determine whether such prioritization improves attendance, capacity utilization, and patient access in real-world practice.

**Abstract:**

Background/Objectives: Patient non-attendance disrupts dental scheduling and may leave reserved appointment time unused when missed slots cannot be reassigned. Most no-show prediction studies focus on discrimination or the number of missed appointments identified, without considering appointment duration, probability calibration, temporal robustness, or limited intervention capacity. This study developed and evaluated a calibrated, duration-weighted framework for operational prioritization of dental appointments. Methods: A retrospective secondary analysis was conducted using 196,018 appointments recorded in 2019 at a dental clinic in Riyadh, Saudi Arabia. L2-regularized logistic regression was used as the primary model, with CatBoost as a secondary nonlinear comparator. Model development, probability calibration, and testing were performed in separate chronological periods. Performance was assessed using ROC-AUC, average precision, Brier score, calibration measures, rolling-origin temporal validation, dentist-level transportability analysis, and sensitivity analyses. Four prioritization strategies were compared at intervention budgets from 5% to 30%: random, probability-only, duration-only, and calibrated probability multiplied by scheduled duration. Results: In the independent November–December test set, the calibrated logistic model achieved a ROC-AUC of 0.6886 (95% CI: 0.6827–0.6941), average precision of 0.6087, and Brier score of 0.2238. At the prespecified 10% intervention budget, probability-only targeting captured 15.88% of scheduled no-show minutes, duration-only targeting captured 18.49%, and duration-weighted targeting captured 23.04%. Duration-weighted targeting exceeded probability-only targeting by 7.16 percentage points (95% CI: 6.47–7.79) and duration-only targeting by 4.54 percentage points (95% CI: 4.00–5.39). The advantage persisted across rolling-origin validation and sensitivity analyses. Conclusions: Combining calibrated no-show probability with scheduled appointment duration identified more scheduled time associated with non-attendance than either probability or duration alone under the same intervention budget. The framework demonstrated a retrospective ranking advantage for identifying scheduled minutes associated with non-attendance but did not establish recoverable clinical capacity, reductions in missed appointments, or improvements in clinical efficiency. Independent external validation and prospective intervention studies are required before routine clinical implementation.

## 1. Introduction

Patient non-attendance, commonly described as a missed appointment or no-show, remains a persistent challenge for outpatient healthcare delivery. A no-show occurs when a patient does not attend a scheduled appointment and does not cancel sufficiently early for the time slot to be reassigned. Although the immediate consequence is an unused appointment, the broader effects include disrupted clinical workflows, inefficient use of professional time, reduced service capacity, longer waiting lists, and delayed access for other patients. In dental services, these effects may be particularly important because appointments frequently reserve procedure-specific blocks of clinical time, require coordination between dentists, dental assistants, equipment, and treatment rooms, and may form part of a multistage treatment plan [1,2].

Non-attendance also has consequences beyond operational efficiency. Missed dental visits can interrupt treatment continuity, postpone preventive or restorative care, and increase the likelihood that patients return later with more complex needs. In university dental clinics, failed attendance additionally results in lost clinical teaching opportunities, while in publicly funded or high-demand services, it prevents available capacity from being offered to patients awaiting treatment [1,2]. Previous dental studies have associated attendance behaviour with patient age, socioeconomic characteristics, appointment timing, treatment category, booking lead time, appointment confirmation, and previous attendance patterns [1,2,3]. These associations, however, vary substantially across clinical settings, patient populations, and scheduling systems, limiting the usefulness of uniform rules applied to all dental appointments.

Reminder systems are among the most frequently used strategies for reducing missed appointments. Evidence from dental clinics suggests that short-message reminders can reduce failures to attend, although their effect is not uniform and reminders may also increase timely cancellations, thereby allowing clinics to reallocate appointments [2]. Universal reminders are relatively easy to automate, but more resource-intensive actions—such as live telephone calls, patient-navigation support, active confirmation, or targeted rescheduling—cannot always be applied to every appointment. Dental practices therefore face a practical allocation problem: when administrative capacity is limited, which appointments should receive additional intervention?

Predictive modelling has increasingly been proposed as a solution to this problem. A systematic review of healthcare no-show prediction studies showed that logistic regression, decision trees, random forests, boosting methods, support vector machines, neural networks, and other machine-learning approaches have been used to estimate the probability of non-attendance [3]. Across healthcare settings, previous attendance history and booking lead time have repeatedly emerged as influential predictors, although reported model performance and validation strategies have been heterogeneous [3].

Recent research has examined prediction specifically in dental services. Alabdulkarim et al. analysed 196,018 dental appointments and developed machine-learning models incorporating demographic, scheduling, weather, and prior-attendance information. Their best-performing model achieved a receiver operating characteristic area under the curve of 0.718, and the authors proposed a sequential representation of previous attendance behaviour [4]. Cuevas-Nunez et al. subsequently evaluated eight machine-learning algorithms using electronic health record data from dental school clinics and developed a user-oriented risk score, with their best model attaining an area under the curve of 0.76 [5]. In a multicentre Saudi Arabian study, Almutairi and Olatunji compared decision trees, random forests, and a multilayer perceptron across five dental facilities and nine dental specialties, reporting areas under the curve of approximately 0.83–0.84 and using explainable artificial intelligence methods to interpret predictor contributions [6].

These studies demonstrate that dental no-show risk can be estimated from routinely collected administrative and clinical information. Nevertheless, a recent systematic review of machine-learning applications for dental appointment no-shows identified persistent limitations, including single-centre development, inconsistent outcome and performance reporting, restricted external validation, and little evidence of real-world implementation [7]. Most existing studies have concentrated on algorithm comparison, feature importance, classification accuracy, F1 score, or area under the receiver operating characteristic curve. These measures are useful for describing discrimination, but they do not determine whether predicted probabilities are sufficiently reliable to guide operational decisions or whether acting on those predictions produces meaningful management benefits.

This distinction is important because discrimination and operational value are not equivalent. A model may rank high-risk appointments reasonably well while producing poorly calibrated probabilities. If an appointment is assigned a predicted no-show risk of 60%, clinic managers need to know whether approximately 60% of comparable appointments are actually missed. Otherwise, thresholds for telephone reminders, active confirmation, wait-list activation, or overbooking may be inappropriate. Current guidance for prediction-model research therefore emphasises the assessment of calibration, validation under realistic conditions, transparent reporting, and explicit consideration of the model’s intended use rather than reliance on discrimination alone [8].

The conventional unit of analysis in no-show prediction is the individual appointment, and each missed appointment is generally assigned equal importance. From an operational perspective, however, a missed 60 min appointment is not equivalent to a missed 10- or 15 min appointment. Ranking appointments solely according to no-show probability may maximise the number of non-attenders identified but fail to protect the greatest amount of scheduled clinical capacity. Conversely, incorporating appointment duration into the prioritisation rule may identify fewer individual no-shows while capturing more scheduled time exposed to non-attendance.

Healthcare operations studies have shown that predicted no-show risk can be linked to targeted reminders, patient navigation, dynamic overbooking, and appointment-scheduling policies [9,10,11,12]. A rapid systematic review found that predictive model-based text reminders, telephone calls, and patient-navigation interventions can reduce non-attendance, but evidence regarding overbooking, cost-effectiveness, acceptability, and equity remains limited [9]. A randomised quality-improvement study also demonstrated that model-guided live telephone outreach produced a modest reduction in overall no-show rates and reduced attendance disparities for Black patients in a primary-care setting [10]. Other studies have incorporated individual predicted risk into dynamic overbooking strategies or combined no-show and appointment-length predictions to reduce clinician idle time and patient waiting [11,12]. However, these management-oriented approaches have rarely been evaluated in dentistry, where scheduled appointment durations can vary considerably and where the consequences of unused time depend on the length and organisation of the reserved treatment block.

A further methodological concern is whether model performance persists when predictions are applied to future appointments or to dentists whose activity patterns differ from those represented in model development. Random train–test splitting can place highly similar appointments from the same period, provider, or duplicated administrative records in both datasets, potentially producing optimistic estimates. For a model intended to guide routine scheduling, temporal validation and provider-level transportability are more informative because they approximate the practical questions faced by a clinic: will the model remain reliable next month, and will it perform consistently across different dentists?

The availability of the public dataset reported by Alabdulkarim et al. provides an opportunity to address these questions [4]. In addition to its large sample size, the dataset includes appointment outcome, scheduled duration, booking and appointment timestamps, dentist identifier, SMS status, demographic variables, and contextual information. It therefore permits a decision-oriented reanalysis that extends beyond reproducing the original classification task. At the same time, secondary use of public administrative data requires an explicit integrity audit, because missing variables, anonymisation, exact duplicate records, and the absence of patient-level linkage may influence both apparent predictive performance and the interpretation of management outcomes.

Accordingly, the primary aim of this retrospective secondary analysis was to develop and evaluate a calibrated, duration-weighted framework for dental appointment management using 196,018 publicly available appointment records. The specific objectives were: (1) to audit the integrity, completeness, and temporal consistency of the public dataset; (2) to evaluate no-show prediction using realistic temporal and dentist-level validation strategies; (3) to assess both discrimination and probability calibration; (4) to compare conventional prioritisation by predicted no-show probability with prioritisation by expected scheduled minutes at risk, defined as predicted no-show probability multiplied by scheduled appointment duration; and (5) to quantify the operational performance of these strategies when additional intervention can be applied to only a limited proportion of appointments. Sensitivity analyses were planned to examine the influence of exact duplicate records and demographic predictor inclusion.

We hypothesised that duration-weighted prioritisation would identify a greater proportion of scheduled minutes associated with non-attendance than probability-only ranking under the same intervention budget, even if it did not identify the greatest absolute number of individual no-shows. This approach reframes dental no-show modelling from a classification problem into a resource-allocation problem and provides an operationally interpretable basis for targeted confirmation, reminder, and appointment-recovery strategies.

## 2. Materials and Methods

### 2.1. Study Design and Data Source

This study was a retrospective secondary analysis of a publicly available dental appointment dataset originally reported by Alabdulkarim et al. [4]. The source study reported that the appointment data were collected from a local dental clinic in Riyadh, Saudi Arabia, over the 2019 calendar year [4]. The dataset was obtained from the Supplementary File S4 accompanying the original publication and was provided as the comma-separated file peerj-cs-08-1147-s001.csv.

The public dataset contained 196,018 appointment records scheduled between 1 January and 31 December 2019. Each row represented an appointment-level administrative record. The available variables included patient age, sex, marital status, nationality, appointment date and time, booking date and time, scheduled appointment duration, SMS status, dentist identifier, attendance status, temperature, and weather conditions.

The public file did not contain a patient identifier, treatment type, dental specialty, chair identifier, actual treatment duration, or financial information. Consequently, the analysis was performed at the appointment level, and repeated appointments belonging to the same patient could not be identified or grouped.

All records in the published dataset were retained in the primary analysis. No a priori sample-size calculation was performed because the sample size was determined by the complete publicly available dataset.

**Figure 1:**
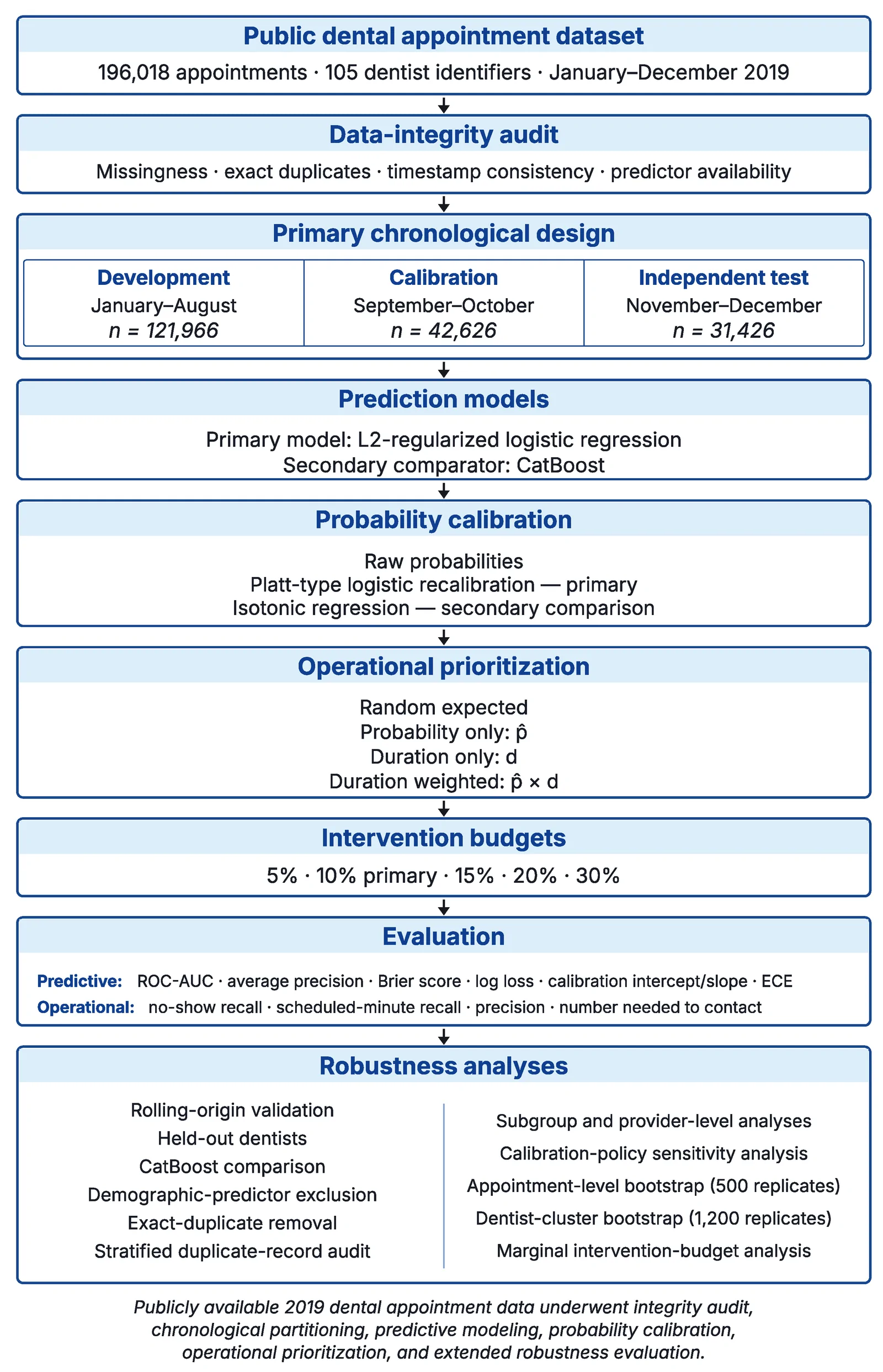
Study design and analytical workflow. The publicly available 2019 dental appointment dataset (196,018 records) underwent a structured data-integrity audit and chronological partitioning into development (January–August), calibration (September–October), and independent temporal test (November–December) sets. L2-regularized logistic regression was used as the primary prediction model, with CatBoost as a secondary nonlinear comparator. Platt-calibrated probabilities were used to compare random, probability-only, duration-only, and duration-weighted prioritization strategies across intervention budgets ranging from 5% to 30%. The evaluation included predictive discrimination, probability calibration, and scheduled-minute recall. Robustness assessments comprised rolling-origin temporal validation, held-out-dentist testing, subgroup and provider-level analyses, demographic-predictor exclusion, exact-duplicate removal, and 500 appointment-level bootstrap replicates. Additional post hoc analyses included a stratified duplicate-record audit, calibration-policy sensitivity analysis, 1200 dentist-cluster bootstrap replicates, and marginal intervention-budget analysis.

The overall study design and analytical workflow are summarized in Figure 1.

### 2.2. Data Integrity Audit

Before model development, the dataset underwent a structured integrity audit. The audit examined:

- The number of rows and columns;
- The appointment-date range;
- The number and proportion of no-shows;
- The number of unique dentist identifiers;
- The distribution and range of scheduled appointment duration;
- Missingness in nationality;
- Exact duplicate records;
- Temporal consistency between booking and appointment timestamps;
- The availability of variables at the intended prediction time.

Exact duplicate records were defined as rows with identical values across all 13 published columns. Because the public dataset did not contain a patient or encounter identifier that could distinguish administrative duplication from genuinely separate but observationally identical appointments, duplicate records were retained in the primary analysis. A sensitivity analysis was subsequently conducted after removing exact duplicates. An additional post hoc integrity audit examined the distribution of exact duplicate occurrences across the chronological development, calibration, and test periods, calendar months, dentist identifiers, and scheduled appointment hours. Duplicate occurrences were identified using equality across all 13 published variables, with all occurrences beyond the first identical record flagged as duplicates. The audit was descriptive and did not assume that identical records necessarily represented erroneous administrative duplication.

Appointment and booking timestamps were parsed using day-first date formatting. The appointment date and appointment time fields were combined into a single appointment timestamp. Booking lead time was calculated as:

(1) $$L_{i}=\frac{T_{\mathrm{appointment},i}-T_{\mathrm{booking},i}}{24\;h}$$

where *L_i_* represents the number of days between booking and the scheduled appointment. Negative lead-time values were treated as invalid and set as missing.

Because lead time was right-skewed, it was transformed as:

(2) $$L_{i}^{*}=\ln\left(1+L_{i}\right)$$

### 2.3. Outcome Definition

The binary outcome was appointment non-attendance. The original variable Attend was recoded as:

(3) $$Y_{i}=\left\{\begin{array}{l} 1,\mathrm{if the patient did not attend the appointment}\\ 0,\;\mathrm{if the patient attended the appointment} \end{array}\right.$$

The analysis therefore predicted the probability that a scheduled appointment would result in a no-show.

Scheduled appointment duration was not treated as a measure of actual chair utilization. The dataset did not indicate whether unused appointment time was subsequently reassigned or whether several treatment chairs operated in parallel. Accordingly, operational outcomes were described as scheduled minutes associated with no-shows, rather than confirmed lost chair time.

### 2.4. Predictor Variables

The primary model included information that could reasonably be available before the scheduled appointment.

SMS status, observed temperature, and weather were not included in the primary prediction model. SMS status may reflect an administrative intervention rather than a baseline patient characteristic, while observed weather and temperature may not be known at the time when an appointment-management decision is made. Their exclusion reduced the risk of using information that was unavailable, intervention-dependent, or temporally ambiguous at the intended prediction point.

The continuous predictors were:

- Scheduled appointment duration, in minutes;
- Patient age;
- Log-transformed booking lead time;
- Appointment hour;
- Day of the year.

The categorical predictors were:

- Weekday;
- Calendar month;
- Sex;
- Marital status;
- Nationality;
- Dentist identifier.

Appointment hour was represented as a continuous value incorporating both hours and minutes. Day of the year was included as a continuous representation of progression through the calendar year, while month and weekday were included as categorical variables to capture broader seasonal and weekly scheduling patterns.

A reduced model was also evaluated after excluding sex, marital status, and nationality. Age, appointment characteristics, calendar variables, and dentist identifier were retained. This analysis assessed whether selected categorical demographic attributes materially improved prediction or operational prioritization.

### 2.5. Missing Data and Variable Encoding

Missing categorical values were represented explicitly using a Missing category. Numeric missing values were imputed using the median estimated from the model-development data.

Numeric variables were standardized using the mean and standard deviation of the development data. Categorical variables were one-hot encoded. Categories occurring fewer than 10 times were handled as infrequent categories by the encoder, and previously unseen categories in calibration or test data were ignored rather than causing prediction failure.

All preprocessing operations were incorporated into the modelling pipeline and fitted exclusively using the corresponding training data. Calibration and test data were not used to estimate imputation values, scaling parameters, or categorical encodings.

### 2.6. Primary Temporal Development, Calibration, and Test Sets

A fixed chronological design was used for the primary analysis to approximate prospective implementation.

Appointments were divided into:

- Development set: 1 January–31 August 2019.
- Calibration set: 1 September–31 October 2019.
- Temporal test set: 1 November–31 December 2019.

This produced 121,966 development records, 42,626 calibration records, and 31,426 test records.

The prediction models were fitted using only the development period. Probability-calibration models were fitted using the independent September–October calibration period. All final discrimination, calibration, and operational-policy comparisons were performed on the untouched November–December test period.

A random train–test split was not used for the primary analysis because it would not reproduce the intended use case in which historical appointments are used to predict future appointment behaviour.

### 2.7. Primary Prediction Model

The primary model was an L2-regularized logistic regression implemented as a unified preprocessing and modeling pipeline [13]. The inverse regularization strength was fixed at C = 1.0.

The model was fitted using the Newton–Cholesky solver, with a maximum of 100 iterations and a fixed random seed of 2026. No data-driven hyperparameter search was performed. Logistic regression was selected as the primary method because it provides a transparent reference model and produces continuous probability estimates suitable for subsequent calibration and operational decision analysis.

The complete fitted-model specification, including the encoded predictor names, logistic-regression coefficients, encoded category levels, numeric-variable imputation and standardization parameters, and model intercept, is provided in Supplementary Table S5. All one-hot encoded indicator variables were retained, and no categorical reference level was dropped. Because the model was L2-regularized and developed for prediction rather than etiological inference, the coefficients were not subjected to hypothesis testing and should not be interpreted as causal effects.

### 2.8. Probability Calibration

Raw predicted probabilities were recalibrated using the independent September–October calibration set. Two methods were evaluated:

1. Logistic recalibration, also referred to as Platt-type scaling;
2. Isotonic regression.

For logistic recalibration, a logistic regression model was fitted using the logit of the raw predicted probability:

(4) logitP(Yi=1)=α+β logitp^iraw

where ${\hat{p}}_{i}^{\mathrm{raw}}$ is the raw predicted no-show probability for appointment *i*, *α* is the calibration intercept, and *β* is the calibration slope.

Probabilities were restricted to the interval 10^−6^ to 1 − 10^−6^ before logit transformation.

Platt-calibrated probabilities were designated for the primary operational analysis because the method is parsimonious, monotonic, and preserves the ranking generated by the underlying model. Isotonic calibration was evaluated as a secondary, more flexible calibration approach [14].

Calibration was assessed using:

- Brier score;
- Logarithmic loss;
- Jointly estimated calibration intercept and slope;
- Expected calibration error using 10 quantile-based probability groups;
- Graphical calibration curves.

The fitted logistic-recalibration intercept and slope are also reported in Supplementary Table S5 to permit reconstruction of the final Platt-calibrated probabilities.

### 2.9. Secondary Nonlinear Model

CatBoost was used as a secondary nonlinear comparator because it can directly process categorical variables and model interactions and nonlinear associations [15].

The CatBoost classifier used:

- A total of 500 boosting iterations;
- Tree depth of 7;
- Learning rate of 0.05;
- L2 leaf regularization of 5;
- Binary logarithmic loss;
- AUC as the evaluation metric;
- Random seed 2026.

Numeric missing values were replaced with development-set medians, and categorical missing values were represented as Missing. The CatBoost model was fitted on the same development period as the primary logistic model. Its probabilities were recalibrated using Platt scaling on the same independent calibration period. CatBoost was treated as a secondary comparator rather than the basis of the primary study conclusions.

### 2.10. Predictive Performance Measures

Discrimination was assessed using:

- Area under the receiver operating characteristic curve;
- Average precision (AP), used as the summary measure of precision–recall performance.

Because no-shows were frequent but not evenly balanced with attended appointments, average precision was reported in addition to ROC-AUC.

Probabilistic accuracy and calibration were assessed using:

- Brier score;
- Logarithmic loss;
- Calibration intercept;
- Calibration slope;
- Expected calibration error;
- Calibration plots.

The evaluation framework followed the principle that prediction models intended to support operational decisions should be assessed for calibration and temporal reliability, rather than discrimination alone [8,14].

### 2.11. Duration-Weighted Operational Prioritization

The primary management analysis examined how predicted risk could be used when a dental service had sufficient administrative capacity to contact or otherwise intervene on only a limited proportion of upcoming appointments.

For each appointment, the expected scheduled minutes at risk (SMAR) were defined as:

(5) $${\mathrm{SMAR}}_{i}={\hat{p}}_{i}\times d_{i}$$

where ${\hat{p}}_{i}$ is the Platt-calibrated probability of a no-show for appointment *i* and *d_i_* is the scheduled duration of appointment *i*, expressed in minutes. Throughout this study, scheduled minutes at risk (SMAR) represents the expected scheduled duration associated with appointment non-attendance, rather than confirmed unused chair time, recoverable clinical capacity, or monetary loss. The score is intended for retrospective prioritization analysis and does not incorporate subsequent slot reassignment, intervention effectiveness, or actual resource utilization.

Four prioritization policies were compared:

1. Random targeting: the analytically expected result of selecting appointments at random.
2. Probability-only targeting: appointments ranked from highest to lowest calibrated no-show probability.
3. Duration-only targeting: appointments ranked from longest to shortest scheduled duration.
4. Duration-weighted targeting: appointments ranked according to ${\hat{p}}_{i}\times d_{i}$.

Appointments were ordered chronologically before operational ranking. For tied ranking scores, the original chronological row order was retained, consistent with the pandas nlargest() procedure used in the analysis notebook. This convention was particularly relevant to duration-only prioritization because multiple appointments shared identical scheduled durations.

The random strategy was represented by its analytical expectation rather than by a single random Monte Carlo selection. This provided a stable reference corresponding to the fraction of appointments selected.

Operational performance was assessed at intervention budgets of: 5%, 10%, 15%, 20% and 30%. For each budget b, the number of appointments selected was:

(6) *k* = ⌈*bN*⌉

where:

- *b* is the intervention-budget proportion;
- *N* is the total number of appointments in the relevant test dataset;
- *k* is the number of appointments selected for intervention;
- The ceiling brackets indicate that the result was rounded upward to the nearest whole appointment.

The 10% intervention budget was used as the primary comparison, representing a scenario in which additional administrative action could be applied to approximately one in every ten appointments. Because the public dataset contained no information on administrative staffing capacity or intervention costs, the 10% budget was specified a priori as an illustrative operational scenario rather than as an empirically optimized threshold. Results across budgets from 5% to 30% were reported to demonstrate how policy performance varied with available intervention capacity.

### 2.12. Operational Performance Measures

For each prioritization strategy, the following outcomes were calculated:

- Number of appointments targeted;
- Number of no-shows captured;
- No-show recall;
- Scheduled minutes associated with no-shows captured;
- Scheduled-minute recall;
- Precision among targeted appointments;
- Number needed to contact to identify one no-show;
- Scheduled no-show minutes captured per 100 targeted appointments.

No-show recall was calculated as:

(7) $$\mathrm{No}-\mathrm{show Recall}=\frac{\mathrm{Number of no}-\mathrm{shows among targeted appointments}}{\mathrm{Total number of no}-\mathrm{shows}}$$

Scheduled-minute recall was calculated as:

(8) $$\mathrm{Scheduled}-\mathrm{Minute Recall}=\frac{\sum_{i\in S}Y_{i}d_{i}}{\sum_{i=1}^{N}Y_{i}d_{i}}$$

where

- *S* denotes the set of appointments selected for intervention;
- *Y_i_* equals 1 when appointment *i* resulted in a no-show and 0 otherwise;
- *d_i_* is the scheduled duration of appointment *i*;
- *N* is the total number of appointments in the evaluation dataset.

The primary operational contrast was the difference in scheduled-minute recall between duration-weighted targeting and probability-only targeting at the 10% intervention budget. A second key contrast compared duration-weighted targeting with duration-only targeting. The latter comparison determined whether any benefit of the proposed method resulted merely from selecting longer appointments.

No assumptions were made regarding the monetary value of appointment time, the causal effectiveness of reminder interventions, or the financial cost of contacting patients. The operational analysis therefore evaluated prioritization yield rather than actual cost savings or causal intervention effects.

As an additional post hoc decision-sensitivity analysis, we calculated the incremental number of scheduled no-show minutes captured per additional appointment selected when expanding the targeting budget. We also considered a hypothetical net-benefit formulation, NB(b) = q × v × M(b) − c × k(b), where M(b) represents scheduled no-show minutes retrospectively captured at budget b, k(b) represents the number of selected appointments, q is an assumed proportion of these minutes that could become productively usable following an effective intervention, v is the assumed value per usable minute, and c is the assumed intervention cost per targeted appointment. None of the intervention-effectiveness or economic parameters was observed in the dataset. Therefore, this expression was used only to illustrate conditional break-even relationships and not to estimate realized economic benefits or an optimal intervention budget.

### 2.13. Rolling-Origin Temporal Validation

Temporal stability was evaluated using rolling-origin validation for each month from July to December 2019.

For each test month:

- All months earlier than the immediately preceding month formed the development dataset;
- The immediately preceding month formed the calibration dataset;
- The next month formed the test dataset.

For example, the July evaluation used January–May for model development, June for probability calibration, and July for testing. The process was repeated sequentially through December.

For every test month, the logistic model and Platt calibration model were refitted. Predictive performance and the four operational policies were then evaluated independently. This analysis assessed whether discrimination, calibration, and the relative advantage of duration-weighted prioritization were consistent over time.

### 2.14. Dentist-Level Transportability

To assess transportability across providers, 20% of the unique dentist identifiers were randomly selected as held-out dentists using random seed 2026. This corresponded to 21 of the 105 dentist identifiers.

The primary logistic model was trained using January–August appointments from the remaining dentists and calibrated using their September–October appointments. Testing was performed using November–December appointments belonging only to the held-out dentists.

Because the held-out dentist identifiers were absent from model development, this analysis assessed performance in providers whose individual scheduling or attendance patterns were not learned by the model. The same predictive and operational performance measures were calculated at the 10% intervention budget.

For appointments from held-out dentists, the previously unseen dentist categories were ignored by the fitted one-hot encoder. Predictions therefore relied on the remaining patient- and appointment-level predictors rather than on learned coefficients specific to those dentists.

### 2.15. Subgroup and Provider-Level Analyses

Model performance was evaluated separately according to:

- Sex;
- Age group;
- Whether nationality was recorded or missing;
- Dentist identifier.

Ages were grouped as follows:

- Patients aged 0–17 years;
- Patients aged 18–34 years;
- Patients aged 35–49 years;
- Patients aged 50–64 years;
- Patients aged 65 years or older.

Subgroup results were reported only when a subgroup contained at least 100 test appointments and both attendance outcomes were present. Dentist-specific results were similarly restricted to dentists with at least 100 appointments in the temporal test set.

For each eligible subgroup, discrimination, calibration, probability-only scheduled-minute recall, and duration-weighted scheduled-minute recall were calculated. These analyses were exploratory and were intended to identify heterogeneity rather than to establish causal demographic effects.

### 2.16. Sensitivity Analyses

Two principal sensitivity analyses were conducted.

First, the entire fixed temporal analysis was repeated after removing all exact duplicate rows from the original CSV file. Because the source data did not permit confirmation that these rows represented erroneous duplication, the analysis using all published rows remained primary.

Second, the logistic model was repeated after excluding sex, marital status, and nationality. This evaluated whether the selected categorical demographic attributes contributed meaningful predictive or operational information.

Raw, Platt-calibrated, and isotonic-calibrated probabilities were also compared to assess sensitivity to the selected calibration method.

A post hoc calibration-policy sensitivity analysis further evaluated whether residual differences in probability calibration changed appointment selection under the proposed duration-weighted strategy. Using the original saved test-set predictions, scheduled-minute recall at the 10% targeting budget was recalculated separately for raw, Platt-calibrated, and isotonic-calibrated probabilities. The proportion of appointments selected in common by Platt-based and isotonic-based duration-weighted ranking was also determined. All calibration models had been fitted exclusively in the independent September–October calibration period, and the November–December test outcomes were used only for evaluation.

### 2.17. Bootstrap Uncertainty Analysis

Uncertainty was quantified using 500 nonparametric bootstrap replicates of the temporal test dataset. In each replicate, appointments were sampled with replacement using a sample size equal to the original test dataset.

The complete operational ranking procedure was repeated within every bootstrap sample. Percentile-based 95% confidence intervals were calculated using the 2.5th and 97.5th percentiles.

Bootstrap confidence intervals were estimated for:

- ROC-AUC;
- Average precision;
- Brier score;
- Scheduled-minute recall for each prioritization strategy at the 10% budget;
- No-show recall for each strategy;
- The difference in scheduled-minute recall between duration-weighted and probability-only targeting;
- The difference in scheduled-minute recall between duration-weighted and duration-only targeting.

Because the public dataset lacked a patient identifier, bootstrap resampling was necessarily performed at the appointment level rather than the patient level. The bootstrap intervals quantify evaluation-sample uncertainty conditional on the fitted prediction and calibration models; model-development uncertainty was not incorporated.

To assess sensitivity to provider-level dependence, an additional post hoc dentist-cluster bootstrap was conducted using the independent November–December 2019 test set. The test set contained 96 unique dentist identifiers. In each of 1200 bootstrap replicates, dentist identifiers were sampled with replacement, and all appointments associated with each sampled dentist were retained. The 10% targeting budget and the probability-only and duration-weighted rankings were recalculated within each replicate. Percentile-based 95% confidence intervals were estimated from the 2.5th and 97.5th percentiles using random seed 2026. This procedure preserved within-dentist clustering but could not account for repeated appointments belonging to the same patient across providers. The intervals remained conditional on the previously fitted prediction and calibration models.

### 2.18. Software and Reproducibility

The analysis was conducted in Google Colab using Python 3.13.5. The principal software libraries were pandas 2.2.3, NumPy 2.3.5, SciPy 1.17.0, scikit-learn 1.8.0, CatBoost 1.2.8, and Matplotlib 3.10.8 [13,15].

A fixed random seed of 2026 was used for bootstrap resampling, selection of held-out dentists, and stochastic model components. The analysis pipeline generated the model-performance tables, operational-budget tables, rolling-origin results, bootstrap confidence intervals, subgroup analyses, test-set predictions, and publication-quality figures automatically.

The complete Google Colab notebook and the generated analysis outputs will be provided as Supplementary Materials to support computational reproducibility.

### 2.19. Ethical Considerations

The present study involved secondary analysis of an anonymized, publicly available appointment-level dataset. No new participants were recruited, no patient contact or clinical intervention occurred, and no attempt was made to re-identify individuals. Therefore, ethical approval was not required for the present secondary analysis.

## 3. Results

### 3.1. Dataset Characteristics and Data-Integrity Audit

The publicly available dataset contained 196,018 dental appointment records scheduled between 1 January and 31 December 2019 and associated with 105 dentist identifiers. A total of 83,663 appointments resulted in non-attendance, corresponding to an overall no-show rate of 42.68%. The dataset represented 7,054,970 scheduled minutes, of which 3,049,845 min, or 43.23%, were associated with no-show appointments.

Scheduled appointment duration ranged from 5 to 300 min, with a median duration of 30 min. Nationality was missing for 54,623 appointments, corresponding to 27.87% of the dataset. The integrity audit identified 39,405 exact duplicate rows beyond the first occurrence, representing 20.10% of all published records. Because the public data did not contain patient or encounter identifiers, these records could not be classified conclusively as erroneous duplicates and were retained in the primary analysis.

The additional duplicate-record audit revealed marked heterogeneity across the chronological data partitions. Of the 39,405 exact duplicate occurrences, 27,211 were present in the January–August development period (22.31% of development records), 11,554 in the September–October calibration period (27.11% of calibration records), and 640 in the November–December test period (2.04% of test records). Thus, 38,765 duplicate occurrences were attributable to January–October 2019, representing 23.55% of records in that combined period. Duplicate occurrences were identified among 71 of the 105 dentist identifiers. Five dentists (IDs 18, 27, 19, 10, and 7) accounted for 13,516 duplicate occurrences (34.30% of all duplicate occurrences), while representing 19.08% of all published appointment records. Regarding appointment time, 22,631 duplicate occurrences (57.43%) involved appointments scheduled between 17:00 and 20:59; however, this interval also represented 57.51% of all appointments. The uneven distribution of duplicate occurrences, especially across model-development and calibration periods, may have influenced observation weighting, fitted predictor relationships, and probability calibration. The anonymized public dataset does not permit definitive identification of the underlying causes of these repeated records. Detailed audit results are provided in Supplementary Table S6A–D.

The fixed temporal design included 121,966 appointments in the January–August development set, 42,626 appointments in the September–October calibration set, and 31,426 appointments in the November–December test set. The temporal test set contained 13,772 no-shows, corresponding to a prevalence of 43.82%, and 504,815 scheduled minutes associated with non-attendance.

The principal dataset characteristics, integrity-audit findings, and chronological data partitions are summarized in Table 1.

### 3.2. Predictive Performance and Probability Calibration

**Figure 2:**
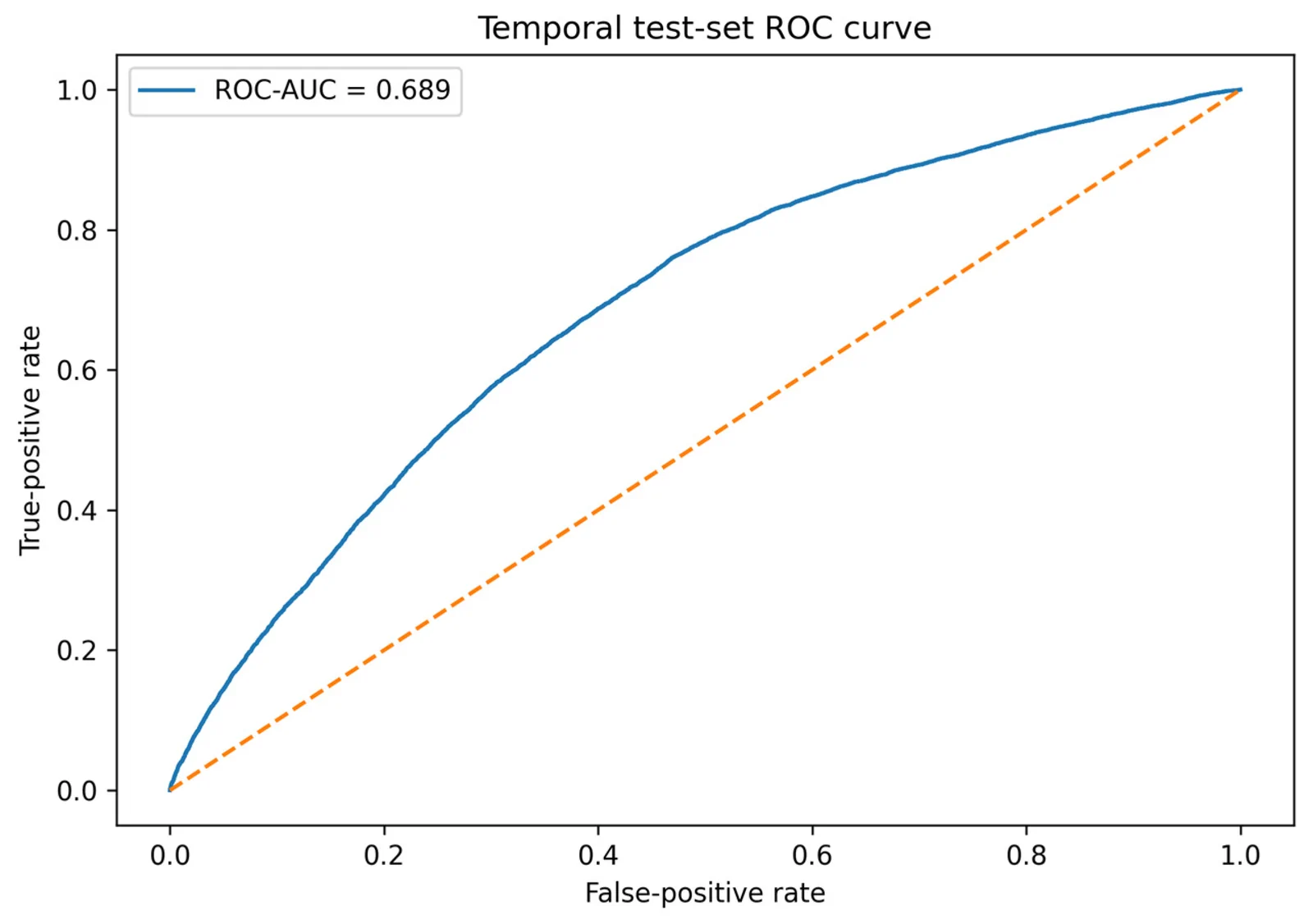
Receiver operating characteristic curve of the Platt-calibrated logistic regression model in the independent November–December 2019 temporal test set. The model achieved a ROC-AUC of 0.6886. The dashed diagonal line represents chance-level discrimination.

**Figure 3:**
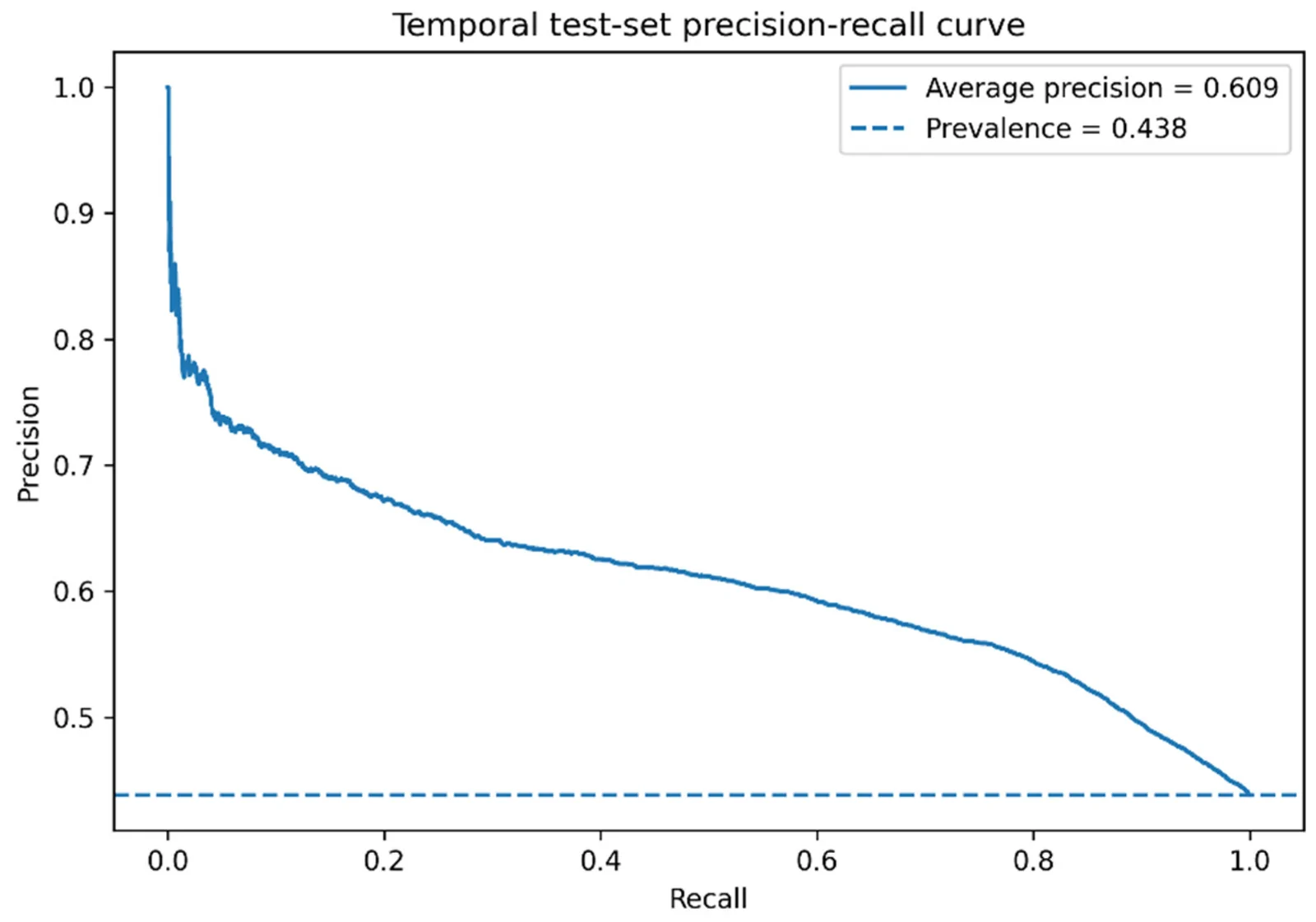
Precision–recall curve of the Platt-calibrated logistic regression model in the independent November–December 2019 temporal test set. Average precision was 0.6087, and the no-show prevalence was 43.82%. The dashed horizontal line represents the prevalence-based reference level.

In the independent temporal test set, the primary logistic regression model achieved a ROC-AUC of 0.6886, with a 95% bootstrap confidence interval of 0.6827–0.6941. Average precision was 0.6087, with a 95% confidence interval of 0.6001–0.6169. The receiver operating characteristic and precision–recall curves are shown in Figure 2 and Figure 3, respectively.

The raw logistic-regression probabilities had a Brier score of 0.2428, a logarithmic loss of 0.6848, and an expected calibration error of 0.1470. Platt calibration reduced the Brier score to 0.2238, the logarithmic loss to 0.6382, and the expected calibration error to 0.0568. For the Platt-calibrated model, the calibration intercept and slope in the temporal test set were 0.301 and 1.089, respectively.

**Figure 4:**
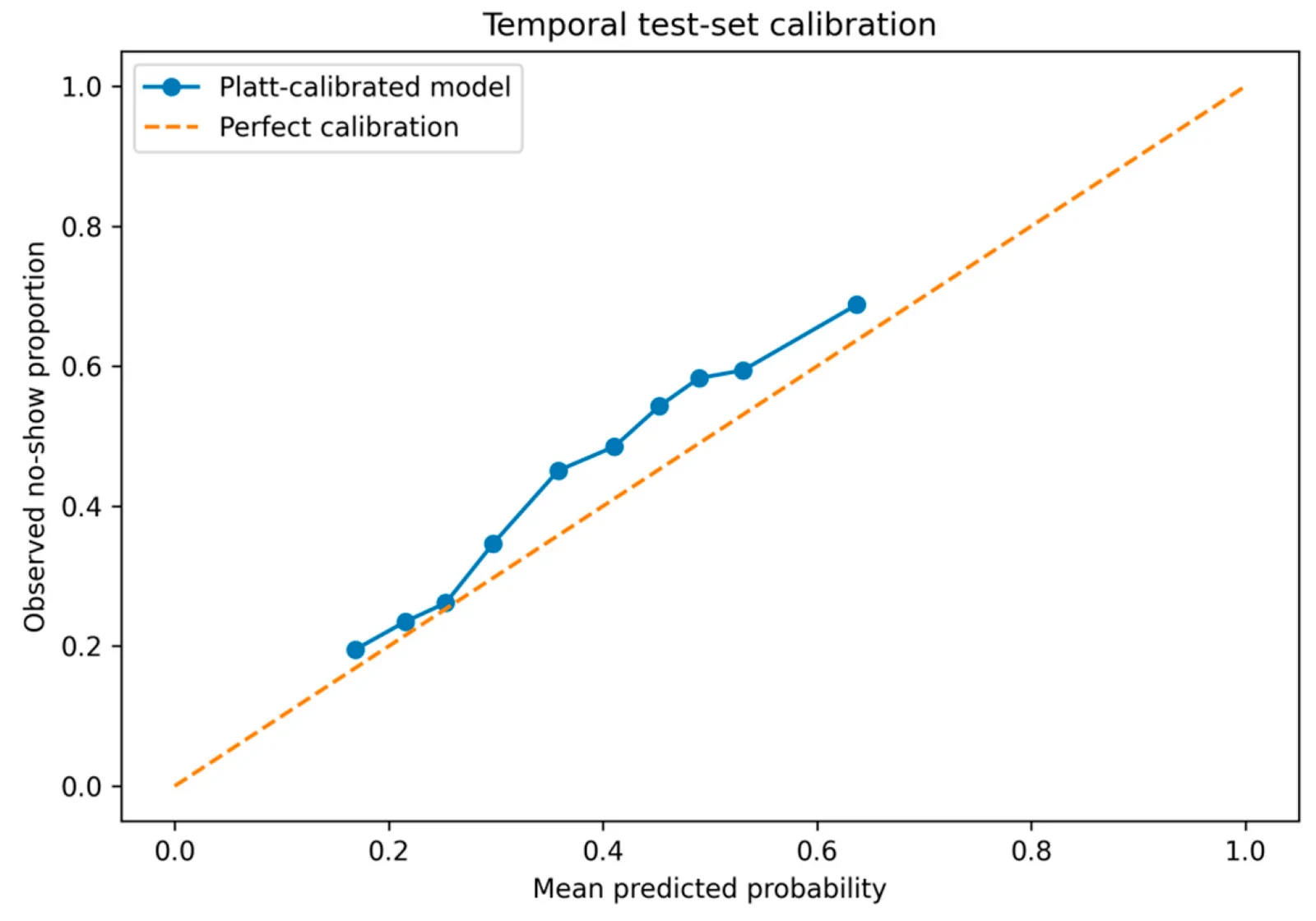
Calibration plot of the Platt-calibrated logistic regression model in the independent temporal test set. Points represent observed no-show proportions across quantile-based groups of predicted probability. The dashed diagonal line represents perfect calibration.

Isotonic calibration produced a Brier score of 0.2232 and an expected calibration error of 0.0524. Its calibration slope was closer to unity, at 1.009, although discrimination was slightly lower, with a ROC-AUC of 0.6877 and an average precision of 0.5992. Because Platt calibration preserved the original model ranking and achieved similar probabilistic performance, Platt-calibrated probabilities were retained for the primary operational analysis. Calibration performance is illustrated in Figure 4, and the complete predictive-performance results are summarized in Table 2.

Residual calibration error remained after Platt recalibration. In the independent temporal test set, the mean Platt-calibrated predicted no-show probability was 38.14%, compared with an observed no-show prevalence of 43.82%, corresponding to an average underprediction of approximately 5.68 percentage points. In the additional calibration-policy sensitivity analysis, duration-weighted scheduled-minute recall at the 10% targeting budget was 22.83% using raw probabilities, 23.04% using Platt calibration, and 23.12% using isotonic calibration. The Platt- and isotonic-based duration-weighted strategies selected 94.02% of appointments in common at this budget. These results indicate that the retrospective scheduled-minute ranking was relatively stable across the evaluated calibration approaches, despite residual absolute-risk miscalibration. Detailed results are provided in Supplementary Table S7.

### 3.3. Operational Performance at the 10% Intervention Budget

At the primary intervention budget of 10%, 3143 of the 31,426 test appointments were selected for additional administrative action. The four prioritization strategies produced markedly different operational profiles.

Probability-only targeting identified 2162 no-shows, corresponding to a no-show recall of 15.70%. These appointments accounted for 80,145 scheduled no-show minutes, corresponding to a scheduled-minute recall of 15.88%. Precision among the targeted appointments was 68.79%, and the number needed to contact to identify one no-show was 1.45.

Duration-only targeting identified 1391 no-shows, corresponding to a no-show recall of 10.10%. However, because this strategy selected the longest appointments, it captured 93,365 scheduled no-show minutes, corresponding to a scheduled-minute recall of 18.49%. Precision was 44.26%, and the number needed to contact was 2.26.

Duration-weighted targeting identified 1869 no-shows, corresponding to a no-show recall of 13.57%. Although this was lower than the no-show recall obtained through probability-only targeting, the selected appointments accounted for 116,290 scheduled no-show minutes, corresponding to a scheduled-minute recall of 23.04%. Precision was 59.47%, and the number needed to contact was 1.68.

Thus, compared with probability-only targeting, duration-weighted targeting captured an additional 36,145 scheduled no-show minutes using the same number of targeted appointments. The observed increase in scheduled-minute recall was 7.16 percentage points, with a 95% bootstrap confidence interval of 6.47–7.79 percentage points. The additional dentist-cluster bootstrap supported the robustness of the primary retrospective comparison. Based on 1200 dentist-cluster bootstrap replicates, the 95% percentile confidence interval for the difference in scheduled-minute recall between duration-weighted and probability-only prioritization was 5.89–8.35 percentage points. The corresponding 95% confidence interval for duration-weighted scheduled-minute recall was 21.67–24.33%. These intervals were wider than the original appointment-level bootstrap intervals, reflecting additional uncertainty under provider-level clustering. Nevertheless, the estimated difference remained positive. The complete clustered-bootstrap results are presented in Supplementary Table S8.

Compared with duration-only targeting, duration-weighted targeting captured an additional 22,925 scheduled no-show minutes. The observed increase in scheduled-minute recall was 4.54 percentage points, with a 95% bootstrap confidence interval of 4.00–5.39 percentage points.

The corresponding percentile-based 95% bootstrap confidence intervals are reported in Table 3.

These findings indicate that the advantage of duration-weighted targeting was not attributable solely to selecting longer appointments.

Thus, targeting 10% of appointments using the duration-weighted strategy captured more than twice the proportion of scheduled no-show minutes expected under random selection.

Operational performance at the prespecified 10% intervention budget is summarized in Table 3.

### 3.4. Operational Performance Across Intervention Budgets

The benefit of duration-weighted prioritization was observed across all evaluated intervention budgets. When only 5% of appointments were targeted, duration-weighted prioritization captured 12.84% of scheduled no-show minutes, compared with 8.18% for probability-only targeting and 10.15% for duration-only targeting.

At intervention budgets of 15%, 20%, and 30%, duration-weighted prioritization captured 31.75%, 39.15%, and 51.69% of scheduled no-show minutes, respectively. The corresponding values were 23.49%, 30.45%, and 44.36% for probability-only targeting and 26.28%, 34.36%, and 46.73% for duration-only targeting.

**Figure 5:**
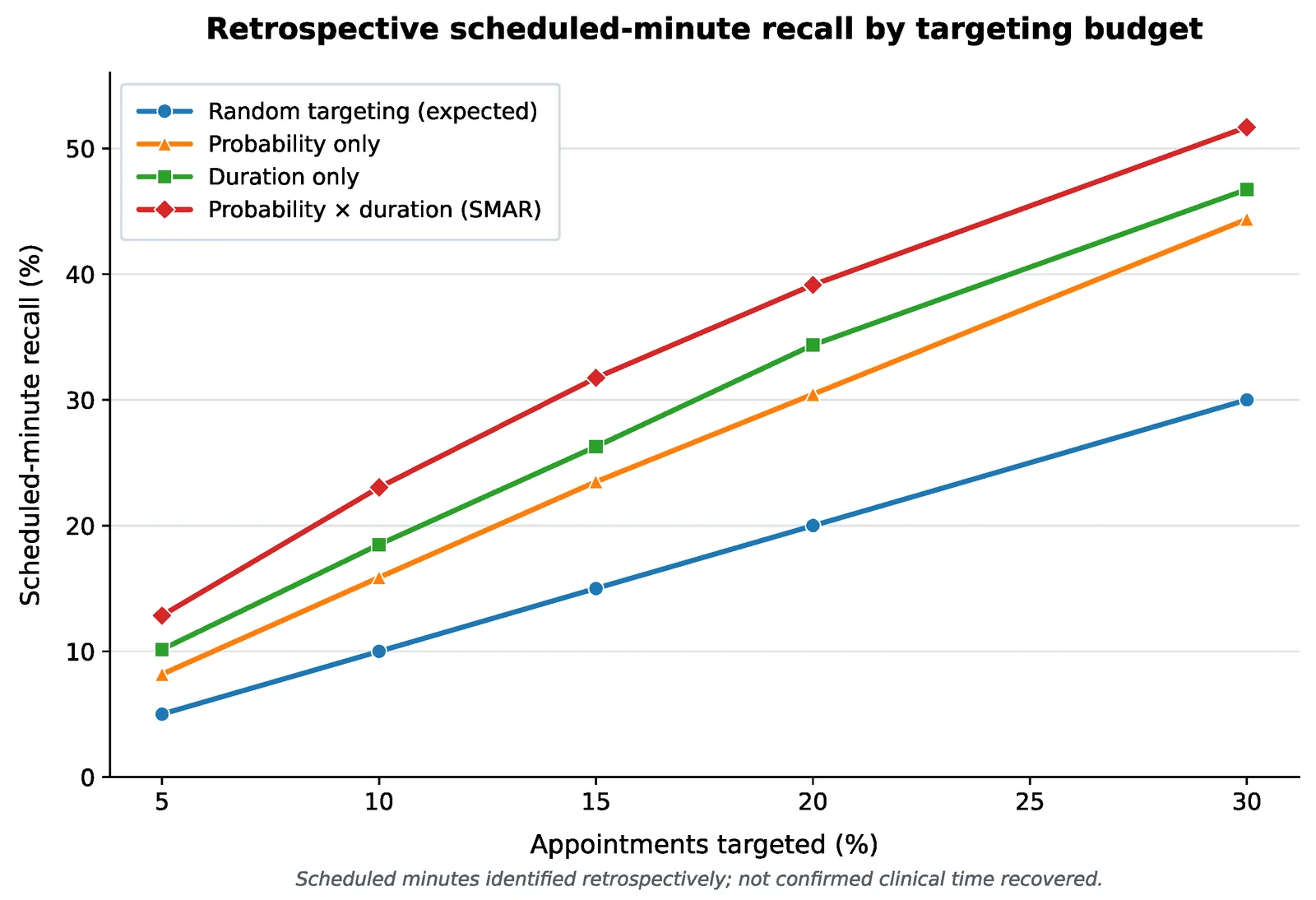
Retrospective scheduled-minute recall across intervention budgets. Scheduled-minute recall in the independent November–December 2019 temporal test set was evaluated at prespecified targeting budgets of 5%, 10%, 15%, 20%, and 30%. Four strategies were compared: random expected targeting, probability-only ranking, duration-only ranking, and duration-weighted ranking based on calibrated no-show probability multiplied by scheduled appointment duration (SMAR). The 10% budget represented the primary comparison. Scheduled-minute recall denotes the proportion of scheduled minutes associated with observed non-attendance captured among selected appointments, not recovered clinical time or demonstrated intervention effectiveness.

Duration-weighted prioritization therefore achieved the highest scheduled-minute recall at every evaluated intervention budget. The relationship between intervention capacity and scheduled-minute recall for all four prioritization strategies is shown in Figure 5. Complete numerical results are provided in Supplementary Table S1.

The post hoc marginal-yield analysis showed decreasing incremental scheduled-minute capture as the targeting budget expanded. The first 5% of selected appointments captured 41.22 scheduled no-show minutes per targeted appointment. Expanding the budget from 5% to 10% captured an additional 32.78 min per additional selected appointment. The corresponding incremental yields were 27.99, 23.77, and 20.15 scheduled no-show minutes per additional appointment for expansions from 10% to 15%, 15% to 20%, and 20% to 30%, respectively. These findings indicate diminishing marginal retrospective selection yield, but they do not establish diminishing causal intervention effectiveness or economic returns. The detailed results are presented in Supplementary Table S9.

### 3.5. Rolling-Origin Temporal Validation

Rolling-origin validation was conducted for each month from July to December 2019. Monthly ROC-AUC values ranged from 0.6805 to 0.7350, with a mean of 0.7044. Average precision ranged from 0.5764 to 0.6707, with a mean of 0.6235, while monthly Brier scores ranged from 0.2018 to 0.2232.

**Figure 6:**
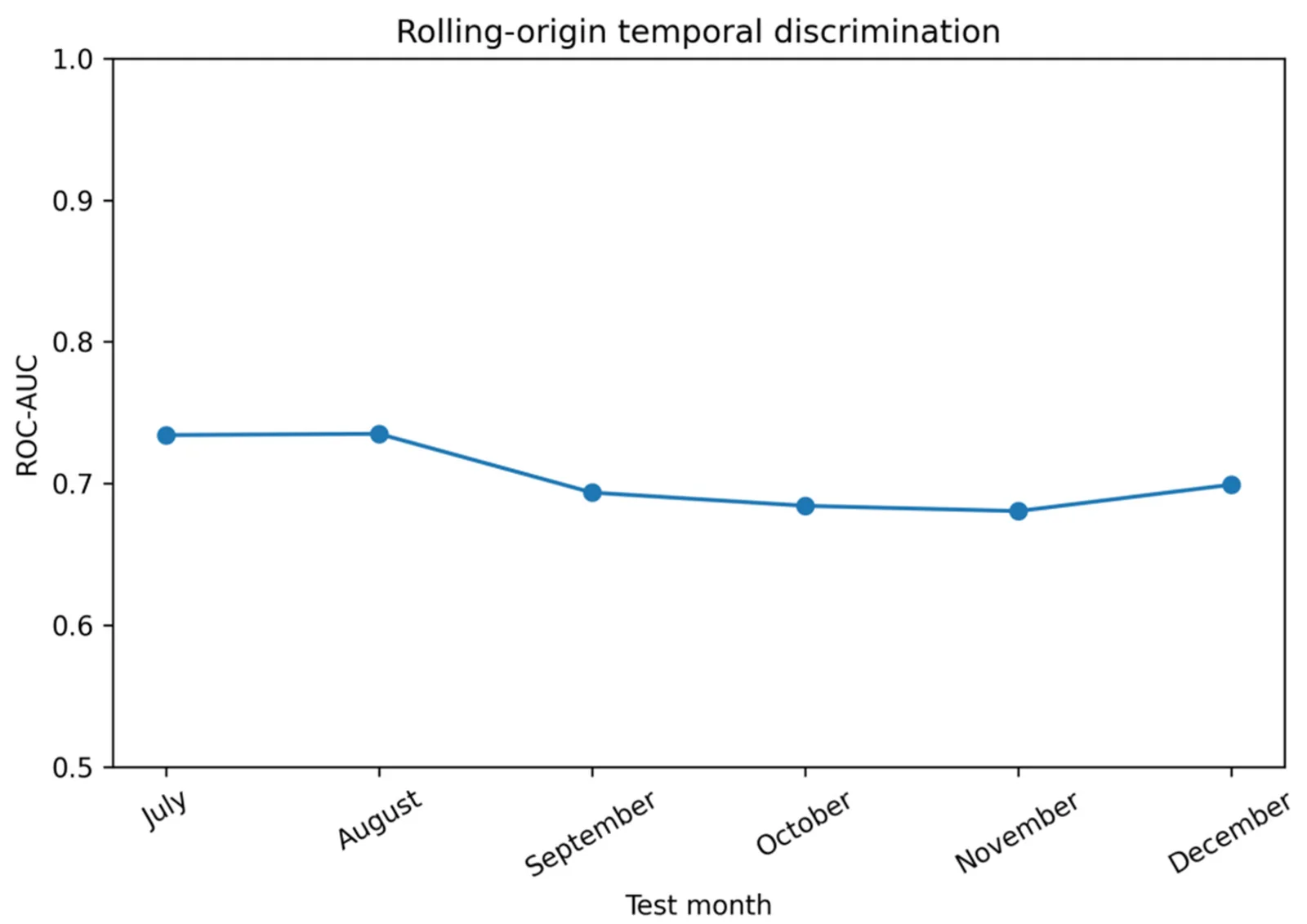
Monthly ROC-AUC during rolling-origin temporal validation from July to December 2019. For each test month, earlier appointments were used for model development, the immediately preceding month was used for probability calibration, and the subsequent month was used for testing.

Calibration remained relatively stable across the six temporal evaluations. The mean calibration intercept was 0.068, the mean calibration slope was 1.005, and the mean expected calibration error was 0.0319. Monthly calibration slopes ranged from 0.816 to 1.114. The monthly ROC-AUC values obtained during rolling-origin temporal validation are presented in Figure 6.

**Figure 7:**
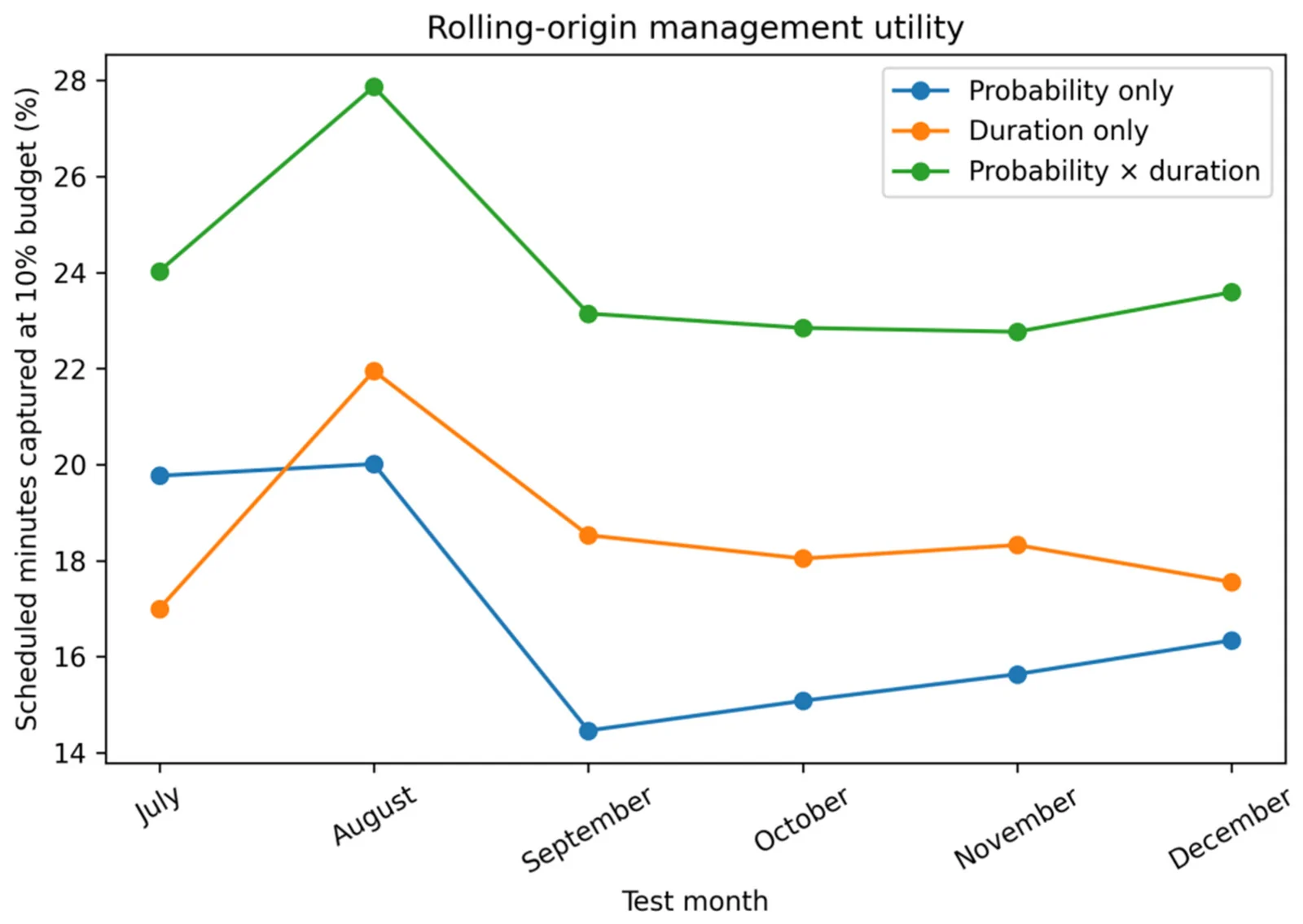
Scheduled-minute recall at the 10% intervention budget during rolling-origin temporal validation for probability-only, duration-only, and duration-weighted prioritization across monthly test periods from July to December 2019.

At the 10% intervention budget, probability-only targeting captured between 14.46% and 20.01% of scheduled no-show minutes across the six monthly test periods, with a mean of 16.88%. Duration-only targeting captured between 17.00% and 21.95%, with a mean of 18.56%. Duration-weighted targeting captured between 22.76% and 27.87%, with a mean of 24.04%. Monthly operational performance is illustrated in Figure 7, while the complete predictive and operational results are summarized in Table 4.

Duration-weighted targeting exceeded probability-only targeting in every monthly test period, with improvements in scheduled-minute recall ranging from 4.26 to 8.69 percentage points. It also exceeded duration-only targeting in every month, with improvements ranging from 4.44 to 7.03 percentage points. These findings demonstrate that the operational advantage of duration-weighted prioritization was temporally consistent and was not attributable solely to the selection of longer appointments.

### 3.6. Secondary Model Analysis

The Platt-calibrated CatBoost model achieved a ROC-AUC of 0.6873 and an average precision of 0.6009. Its Brier score was 0.2212, logarithmic loss was 0.6326, and expected calibration error was 0.0241. The calibration intercept was 0.102 and the calibration slope was 0.999.

CatBoost therefore showed slightly better probabilistic calibration than the logistic model but did not improve discrimination. At the 10% intervention budget, CatBoost probability-only targeting captured 17.54% of scheduled no-show minutes, while CatBoost duration-weighted targeting captured 23.21%. The duration-weighted operational result was therefore very similar to that obtained using the primary logistic regression model.

These findings did not support replacing the more transparent logistic regression model with the more complex nonlinear comparator.

### 3.7. Model Without Selected Demographic Predictors

Excluding sex, marital status, and nationality produced virtually unchanged predictive performance. The Platt-calibrated reduced model achieved a ROC-AUC of 0.6886, an average precision of 0.6086, and a Brier score of 0.2238.

At the 10% intervention budget, the reduced model captured 15.83% of scheduled no-show minutes through probability-only targeting and 22.98% through duration-weighted targeting. These results were almost identical to those of the full model, indicating that the selected categorical demographic variables provided no material improvement in predictive or operational performance.

### 3.8. Dentist-Level Transportability

The dentist-level transportability analysis included 21 held-out dentist identifiers that were not represented during model development or calibration. The corresponding test set contained 7338 appointments and had a no-show prevalence of 41.44%.

Performance decreased when the model was applied to previously unseen dentists. ROC-AUC was 0.6372, average precision was 0.5186, and the Brier score was 0.2298. The calibration slope decreased to 0.780, indicating reduced transportability across providers.

Despite the reduction in predictive performance, duration-weighted targeting retained an operational advantage. At the 10% intervention budget, probability-only targeting captured 12.17% of scheduled no-show minutes, duration-only targeting captured 19.76%, and duration-weighted targeting captured 22.24%.

Duration-weighted targeting also captured 24,155 scheduled no-show minutes, compared with 13,220 min through probability-only targeting and 21,465 min through duration-only targeting.

### 3.9. Subgroup and Provider-Level Performance

Predictive performance was similar between female and male patients. ROC-AUC was 0.6835 among female patients and 0.6904 among male patients. Duration-weighted scheduled-minute recall at the 10% intervention budget was 23.22% and 22.91%, respectively.

Across age groups, ROC-AUC ranged from 0.6590 among patients aged 0–17 years to 0.7267 among patients aged 65 years or older. Duration-weighted scheduled-minute recall ranged from 22.06% to 24.77%. The operational advantage of duration-weighted prioritization was therefore retained in each age category.

Results were also similar according to nationality-data availability. ROC-AUC was 0.6909 among appointments with missing nationality and 0.6872 among those with recorded nationality. Duration-weighted scheduled-minute recall was 23.16% and 22.95%, respectively.

A total of 85 dentist identifiers had at least 100 appointments and both outcome classes in the temporal test set. Dentist-specific ROC-AUC values ranged from 0.5528 to 0.7843, with a median of 0.6803, demonstrating substantial provider-level heterogeneity. Duration-weighted targeting produced higher scheduled-minute recall than probability-only targeting for 68 of the 85 eligible dentists, equal recall for 11 dentists, and lower recall for 6 dentists.

### 3.10. Sensitivity Analysis After Removing Exact Duplicates

After removal of the 39,405 exact duplicate rows, 156,613 appointments remained. The sensitivity-analysis temporal test set contained 30,786 appointments.

Predictive performance remained essentially unchanged. The Platt-calibrated logistic model achieved a ROC-AUC of 0.6893, an average precision of 0.6084, and a Brier score of 0.2232.

At the 10% intervention budget, probability-only targeting captured 15.97% of scheduled no-show minutes, duration-only targeting captured 18.49%, and duration-weighted targeting captured 23.01%. Duration-weighted targeting also identified 13.55% of no-shows, compared with 15.63% for probability-only targeting and 10.11% for duration-only targeting.

The near-identical primary and sensitivity results indicate that the central operational finding was not driven by the exact duplicate records.

The principal secondary, transportability, and sensitivity analyses are summarized in Table 5. Detailed subgroup and dentist-level results are provided in Supplementary Tables S2 and S3.

## 4. Discussion

### 4.1. Principal Findings

This study developed and evaluated a calibrated, duration-weighted framework for translating dental no-show predictions into operational appointment priorities. At the prespecified 10% intervention budget, duration-weighted targeting captured 23.04% of scheduled no-show minutes, compared with 15.88% for probability-only targeting and 18.49% for duration-only targeting. The combined strategy therefore achieved greater retrospective scheduled-minute capture than either predicted no-show probability or scheduled appointment duration used alone.

Duration-weighted targeting did not identify the largest number of individual no-shows. Probability-only targeting achieved a no-show recall of 15.70%, compared with 13.57% for duration-weighted targeting. This trade-off reflects the different objectives of the two strategies: probability-only ranking prioritizes appointments most likely to be missed, whereas duration-weighted ranking prioritizes appointments with the greatest expected scheduled-time impact. The preferred approach should therefore depend on whether a dental service aims primarily to identify the largest number of non-attenders or to protect the greatest amount of scheduled capacity.

The operational advantage of duration-weighted prioritization was observed across intervention budgets ranging from 5% to 30% and in all six rolling-origin monthly evaluations. It also remained evident after removal of exact duplicate records, exclusion of selected demographic predictors, and evaluation among dentists who had not been represented during model development or calibration, although overall predictive performance declined in the latter analysis. These findings support the temporal stability and robustness of the central operational result.

Importantly, these findings represent retrospective appointment-prioritization performance, not the results of an implemented clinical intervention. The observed differences quantify scheduled no-show minutes identified by alternative selection policies rather than actual reductions in non-attendance, recovered treatment-chair capacity, or improved clinical efficiency. Whether the observed ranking advantage translates into practical clinical benefits depends on outreach effectiveness, appointment reassignment, available staffing, and downstream operational consequences. These outcomes require independent prospective evaluation.

### 4.2. Operational Interpretation of Duration-Weighted Prioritization

Most no-show prediction studies treat every missed appointment as having equivalent importance. From an appointment-management perspective, however, a missed appointment of 60 min creates a different scheduling consequence from a missed appointment of 10 or 15 min. A high predicted probability of non-attendance does not necessarily imply a high expected operational impact when the scheduled appointment is short. Conversely, a longer appointment with a moderately elevated no-show probability may expose a greater amount of scheduled capacity.

The scheduled-minutes-at-risk score used in this study provides a direct and interpretable representation of this principle. Multiplying the calibrated no-show probability by scheduled appointment duration estimates the expected number of scheduled minutes associated with non-attendance for each upcoming appointment. For example, an appointment with a calibrated no-show probability of 0.40 and a scheduled duration of 60 min would have an expected scheduled-minutes-at-risk value of 24 min. An appointment with a higher no-show probability of 0.60 but a duration of only 15 min would have a corresponding value of 9 min. Probability-only ranking would prioritize the latter appointment, whereas duration-weighted ranking would prioritize the former. Scheduled appointment duration served two conceptually distinct roles. As a predictor, it could contribute to estimation of the probability of non-attendance; as a multiplicative weight, it represented the operational value assigned to the reserved appointment block. The duration-only comparator was included specifically to determine whether the observed benefit merely reflected preferential selection of longer appointments.

This distinction does not imply that the estimated minutes represent confirmed lost chair time. The public dataset did not report whether unused appointments were subsequently reassigned, whether multiple chairs operated simultaneously, or whether the scheduled duration corresponded to actual treatment duration. Accordingly, the score should be interpreted as an appointment-prioritization measure based on scheduled capacity rather than as a direct estimate of financial loss, clinician idle time, or realized resource waste.

Nevertheless, the measure offers a practical management advantage. It is expressed in units that clinic managers can understand and can be applied without requiring a complex optimization system. It also allows intervention capacity to be specified explicitly. A clinic able to conduct additional outreach for only 5%, 10%, or 20% of upcoming appointments can rank appointments by expected scheduled minutes at risk and select the number corresponding to its available administrative resources.

The SMAR formulation is a direct application of expected-value reasoning and is not proposed as a novel mathematical identity, probability estimator, or optimization algorithm. Rather, the methodological contribution of this study lies in explicitly defining and empirically evaluating an appointment-prioritization objective based on scheduled duration exposed to non-attendance, under identical targeting constraints. This evaluation combines independently calibrated probabilities, chronological development and testing, rolling-origin validation, provider-level transportability assessment, and direct comparisons against probability-only and duration-only ranking. The contribution therefore concerns the structured evaluation of a decision-oriented ranking policy rather than mathematical novelty of the probability–duration product. Moreover, SMAR should not be interpreted as an optimal intervention policy when intervention effectiveness, recoverability of appointment time, and intervention costs vary across appointments.

### 4.3. Comparison with Previous No-Show Prediction Research

Previous studies have demonstrated that dental non-attendance can be predicted using demographic, scheduling, clinical, and previous-attendance information [4,5,6,7]. Reported discrimination has varied considerably, with ROC-AUC values ranging from approximately 0.72 in the original study based on the present public dataset to approximately 0.76 in dental-school records and more than 0.80 in some multicenter analyses [4,5,6]. The ROC-AUC of 0.6886 observed in the present temporal test set was lower than several previously reported estimates.

These values should not be interpreted as direct comparisons of algorithmic quality because the studies differed in available predictors, outcome definitions, clinical settings, and validation designs. In particular, the public file used in the present analysis did not include a patient identifier and therefore did not permit reconstruction of individual attendance histories. Previous attendance behavior has repeatedly been identified as one of the strongest predictors of future non-attendance [3,4]. Its absence likely limited the maximum achievable discrimination.

The present study also used a strict chronological design rather than a random record-level split. The model was developed using January–August appointments, calibrated using September–October appointments, and evaluated only in November–December appointments. Random splitting can distribute records from the same time periods, providers, and potentially duplicated administrative observations across both training and test sets. Such designs may estimate performance under record exchangeability but do not fully reproduce the practical task of predicting future appointments.

More importantly, the primary contribution of the present study was not an increase in ROC-AUC. It was the transition from predicting non-attendance as an isolated classification outcome to evaluating how predictions could support a constrained appointment-management decision. Existing dental studies have generally emphasized discrimination, classification accuracy, F1 score, or feature importance [4,5,6,7]. In contrast, the present analysis combined probability calibration, temporal validation, provider-level transportability, explicit intervention budgets, and operational outcomes based on scheduled appointment duration. Recent evidence further highlights the methodological heterogeneity and implementation challenges of machine-learning approaches to appointment non-attendance. In a comprehensive review of 52 publications, Toffaha et al. identified persistent limitations related to data availability, model development, and translation of predictions into actionable healthcare interventions [16]. Similarly, Tuan et al., in a large retrospective study of 1,118,236 appointments across 15 primary-care clinics, reported an AUROC of 0.852 for no-show prediction and highlighted the importance of scheduling lead time and patient-specific characteristics [17]. Although these results cannot be directly compared with those obtained in dental services because of differences in populations, predictors, and validation designs, they reinforce the need to distinguish predictive discrimination from the practical value of appointment-management policies.

The observed ROC-AUC of 0.6886 indicates moderate rather than high discrimination and does not justify autonomous clinical decision-making based on individual predicted risks. Nevertheless, the objective of the present study was to evaluate alternative retrospective prioritization strategies rather than to establish a stand-alone clinical prediction system. At the 10% targeting budget, duration-weighted ranking achieved a no-show precision of 59.47%, compared with an overall no-show prevalence of 43.82%. Probability-only ranking achieved higher precision (68.79%) and identified more individual no-shows, but it captured fewer scheduled minutes associated with non-attendance. This illustrates the trade-off between individual no-show identification and duration-weighted scheduled-minute capture. The findings support further evaluation of this ranking objective but do not establish sufficient predictive reliability for unsupervised deployment, automatic overbooking, or other consequential clinical management decisions.

### 4.4. Probability Calibration, Temporal Stability, and Model Choice

Probability calibration was essential because the proposed operational score used the numerical predicted probability rather than the rank alone. Raw logistic-regression probabilities showed substantial miscalibration, with a Brier score of 0.2428 and an expected calibration error of 0.1470. Platt calibration reduced these values to 0.2238 and 0.0568, respectively. The improvement demonstrates that a model may retain the same discrimination while producing probability estimates that are more appropriate for decision analysis.

Some residual miscalibration remained in the independent temporal test set, as indicated by a calibration intercept of 0.301 and a calibration slope of 1.089. Isotonic regression produced slightly lower Brier score and expected calibration error, but its discrimination was marginally lower. Platt calibration was selected for the primary operational analysis because it was parsimonious, preserved the ranking of the underlying model, and produced performance similar to that of the more flexible isotonic approach.

The additional calibration-policy sensitivity analysis demonstrated relatively small differences in retrospective duration-weighted scheduled-minute recall across raw, Platt-calibrated, and isotonic-calibrated probabilities. The high overlap between appointments selected by Platt- and isotonic-based ranking further suggests that the principal finding was not highly sensitive to the evaluated calibration method within the present test period. However, the residual underprediction of no-show probability remains important. Stable retrospective ranking does not imply reliable absolute-risk estimation for individual appointments, nor does it guarantee ranking stability after changes in attendance prevalence or provider characteristics. Local calibration assessment and updating would therefore be necessary before any prospective implementation.

Rolling-origin validation provided a more demanding evaluation of temporal stability than a single fixed test period. Monthly ROC-AUC values ranged from 0.6805 to 0.7350, while duration-weighted scheduled-minute recall at the 10% intervention budget ranged from 22.76% to 27.87%. Although predictive performance varied over time, duration-weighted targeting outperformed probability-only and duration-only targeting in every evaluated month. This consistency is important because management usefulness depends not only on average predictive performance but also on whether the selected policy retains its advantage as appointment patterns change.

The secondary CatBoost model did not improve discrimination, achieving a ROC-AUC of 0.6873 compared with 0.6886 for logistic regression. CatBoost showed a slightly lower Brier score and expected calibration error after Platt calibration, but its duration-weighted operational performance was almost identical to that of the logistic model. These findings suggest that the principal management gain resulted from the operational prioritization rule rather than from increased algorithmic complexity. In this setting, the greater transparency and simpler implementation of regularized logistic regression supported its selection as the primary model.

Recent methodological guidance further emphasizes the importance of evaluating prediction models beyond discrimination alone. The PROBAST+AI framework introduced by Moons et al. provides updated criteria for assessing model-development quality, risk of bias, and applicability, with particular attention to data sources, predictors, outcomes, and analytical procedures [18]. These considerations are especially relevant to the present study because the source dataset contained substantial exact duplication, lacked patient identifiers, and did not permit independent institutional validation. Although a formal PROBAST+AI assessment was not conducted, its methodological principles reinforce the importance of transparent reporting, calibration assessment, sensitivity analyses, and appropriately restricted claims regarding clinical applicability.

### 4.5. Provider-Level Transportability and Subgroup Performance

Performance declined when the model was evaluated among 21 dentist identifiers that had not been represented during development or calibration. ROC-AUC decreased to 0.6372, average precision to 0.5186, and the calibration slope to 0.780. This performance decline is compatible with differences in dentist-specific scheduling practices, patient case mix, appointment characteristics, or administrative procedures. However, the available data do not allow these potential explanations to be isolated or established as causal determinants of the observed calibration drift.

The decline should not be interpreted solely as model failure. Rather, it demonstrates the importance of evaluating whether a prediction system remains valid when applied to providers whose patterns were not observed during development. An appointment-management model introduced into a new clinic or provider group may require local recalibration, periodic updating, or a development sample that represents a broader range of provider environments.

Before implementation in a new provider population, the model should undergo local chronological validation using previously unseen appointment records. Discrimination, calibration-in-the-large, calibration slope, Brier score, and scheduled-minute recall should be evaluated before operational use. If systematic miscalibration is identified, the original model probabilities may be updated by estimating a new calibration intercept and slope from a separate local calibration period, followed by evaluation in a later, untouched validation period. Provider-specific heterogeneity and temporal changes should also be monitored, with periodic recalibration or model updating when clinically relevant deterioration is observed. These procedures are proposed deployment safeguards and were not implemented or evaluated in the present retrospective study.

Despite the reduction in discrimination, duration-weighted targeting continued to capture more scheduled no-show minutes than the alternative strategies among held-out dentists: 22.24%, compared with 12.17% for probability-only targeting and 19.76% for duration-only targeting. The operational rule therefore retained value even when probability ranking became less accurate.

Subgroup analyses showed relatively similar performance between male and female patients and between appointments with recorded and missing nationality data. The duration-weighted advantage was also retained across all age groups. However, these analyses were exploratory and should not be interpreted as evidence of demographic fairness. Fairness assessment would require more complete information on socioeconomic status, access barriers, treatment needs, intervention receipt, and downstream clinical consequences.

The model excluding sex, marital status, and nationality achieved almost identical predictive and operational performance to the full model. This result suggests that these variables added little practical information in the present dataset and that a reduced implementation could avoid using selected demographic attributes without materially reducing performance. Nevertheless, the finding is dataset-specific and does not establish that demographic information is universally irrelevant to dental attendance behavior.

### 4.6. Implications for Dental Appointment Management

The proposed framework is most relevant when universal automated reminders are already available but more resource-intensive interventions cannot be applied to every appointment. Examples include live telephone confirmation, patient-navigation support, individualized rescheduling, transport assistance, or activation of a waiting list. Previous healthcare studies have shown that model-guided reminders, navigation, and overbooking strategies may reduce non-attendance or improve scheduling efficiency [9,10,11,12]. Recent implementation-oriented research has also examined how predictive models can be integrated into operational appointment-management systems. Toker et al. evaluated an artificial intelligence-based appointment system that combined no-show prediction with substitute appointment allocation and reported improvements in service utilization and hospital operational performance [19]. Their findings illustrate the potential importance of connecting predictive information to actual scheduling interventions. However, the intervention design and clinical setting differed from those of the present study, and these findings should not be interpreted as direct evidence that SMAR-based prioritization improves clinical outcomes. Rather, they reinforce the need for prospective evaluation of the proposed ranking strategy.

In practice, the duration-weighted score could be calculated when the appointment list is generated and displayed within a scheduling dashboard. Staff could then select the highest-ranked appointments according to the number of calls or interventions that can be completed. The intervention budget would not need to remain fixed at 10%; it could be adapted to daily staffing, appointment volume, or the availability of replacement patients.

The intervention budgets evaluated in this study represent analytical constraints on the proportion of appointments selected for additional attention, rather than observed staffing capacities or economically optimized thresholds. Although selecting a greater proportion of appointments increases the cumulative scheduled-minute recall, this does not imply that administrative interventions can be expanded proportionally without additional cost or loss of effectiveness. At higher targeting levels, such as 20% or 30%, clinics may encounter limited staff availability, unsuccessful contact attempts, patient contact fatigue, and diminishing marginal benefits. The economic feasibility of any targeting threshold would depend on intervention costs, contact success rates, the probability that an intervention prevents non-attendance, the proportion of released appointment time that can actually be reassigned, and the value of recovered resources. These quantities were unavailable in the public dataset. Consequently, the present results describe retrospective prioritization performance across hypothetical outreach budgets and should not be interpreted as evidence that any particular budget is operationally optimal or cost-effective. The additional marginal-yield analysis provides a quantitative description of how retrospective scheduled-minute capture changes with targeting capacity. Although marginal capture decreased at higher budgets, this does not identify the economically preferred intervention level. Any such choice would require empirical estimates of intervention effectiveness, recoverable appointment capacity, outreach costs, staffing constraints, and the value of alternative uses of clinical time.

The score may also support differentiated interventions. Appointments with high probability but short duration might receive automated confirmation, whereas appointments with high expected scheduled minutes at risk might receive live outreach or active wait-list preparation. However, the present analysis did not evaluate such tiered policies and did not establish that any specific intervention would causally reduce non-attendance.

Duration-weighted ranking should also not be used automatically to justify overbooking. Overbooking may reduce clinician idle time but can increase patient waiting and staff workload when all scheduled patients attend [11,12]. Prospective implementation should therefore evaluate both benefits and unintended consequences, including patient experience, workflow disruption, equity, and the costs of administrative intervention.

### 4.7. Strengths and Limitations

This study has several strengths. It used a large public dataset containing more than 196,000 dental appointments and conducted an explicit data-integrity audit before model development. Predictor inclusion was evaluated according to availability at the intended decision point, reducing the risk of using intervention-dependent or temporally unavailable information. Model development, probability calibration, and testing were performed in separate chronological periods. The analysis also included rolling-origin validation, held-out-dentist evaluation, bootstrap confidence intervals, a nonlinear comparator, subgroup analyses, and sensitivity analyses addressing exact duplicate records and demographic predictor inclusion.

The primary operational analysis evaluated several prespecified hypothetical intervention budgets and compared probability-only ranking not only with random targeting but also with duration-only targeting. This distinction allowed the analysis to determine whether the observed improvement resulted from combining probability and duration rather than merely from selecting the longest appointments. Nevertheless, the uneven temporal distribution of duplicate occurrences warrants additional caution, particularly because the earlier period supplied the model-development and calibration data.

Several limitations must also be acknowledged. First, the public dataset did not include a patient identifier. Consequently, repeated appointments belonging to the same individual could not be linked, previous attendance histories could not be reconstructed, and patient-level dependencies could not be incorporated into bootstrap resampling. The reported confidence intervals were therefore estimated using appointment-level rather than patient-clustered bootstrap samples. If repeated observations from the same individuals exhibit correlated attendance behavior, treating appointments as independent sampling units may underestimate evaluation-sample variance and produce confidence intervals that are narrower than those obtained using patient-level clustered resampling. Furthermore, the reported intervals are conditional on the fitted prediction and calibration models and do not incorporate uncertainty arising from model development or differences between clinical institutions. The confidence intervals should therefore be interpreted with these restrictions in mind.

A supplementary dentist-cluster bootstrap was performed to assess sensitivity to provider-level dependence. Although the resulting confidence intervals were wider than those based on appointment-level resampling, the estimated scheduled-minute recall advantage remained positive. However, clustering by dentist cannot replace clustering by patient, particularly when individual patients may have appointments with different providers. Furthermore, this supplementary bootstrap did not incorporate model-development or calibration-fitting uncertainty.

Second, 39,405 exact duplicate records were identified. The absence of patient and encounter identifiers prevented determination of whether these represented erroneous duplication or genuinely distinct but observationally identical appointments. Retaining them in the primary analysis preserved the published dataset, while the near-identical results after their removal reduced concern that they drove the principal conclusions.

The additional audit identified substantial variation in duplicate prevalence across development, calibration, and test periods and across dentist identifiers. This uneven distribution could affect observation weighting, fitted predictor relationships, and the effective sample size. Similar results after duplicate removal provide reassurance regarding the primary retrospective finding but do not establish the origin of the repeated records or eliminate all potential data-integrity concerns.

Third, the dataset did not contain treatment type, dental specialty, chair identifier, urgency, actual treatment duration, direct financial costs, or information on whether unused appointment slots were reassigned. Scheduled no-show minutes should consequently not be interpreted as confirmed lost clinical time or monetary loss.

Fourth, the data were obtained from a single underlying service and covered one calendar year. Holding out dentists tested provider-level transportability within that source but did not constitute external validation in an independent dental organization, healthcare system, or country. The observed decline in performance among held-out dentists further indicates that local validation and recalibration would be necessary before implementation elsewhere.

No independent external dental-clinic dataset was available for validation. Therefore, transportability to other institutions, healthcare systems, countries, or later calendar periods has not been established. The held-out-dentist experiment should be interpreted as within-source provider transportability rather than independent external validation. The framework requires external validation and prospective evaluation before routine clinical adoption. Although the general principle of combining calibrated no-show probability with scheduled appointment duration may be applicable across different clinical settings, the magnitude of its operational benefit is likely to depend on local appointment-duration distributions, attendance patterns, and available intervention resources.

Fifth, this was a retrospective policy-evaluation study. It estimated which appointments would have been prioritized but did not apply reminders, rescheduling, navigation, or overbooking interventions. It therefore cannot establish that duration-weighted targeting reduces no-show rates, increases actual chair utilization, improves access, or produces financial savings. The findings concern prioritization yield rather than causal intervention effectiveness.

Sixth, SMAR estimated expected scheduled minutes associated with non-attendance rather than expected preventable minutes. If the effectiveness of outreach varies across patients, appointment types, or providers, an optimal intervention policy would also require estimates of heterogeneous intervention effects and intervention costs. Such information was unavailable in the public dataset.

### 4.8. Future Research

Future studies should prospectively compare probability-only and duration-weighted prioritization within routine dental scheduling. A cluster-randomized or stepped-wedge design could evaluate whether model-guided outreach reduces non-attendance and increases the reuse of scheduled capacity without increasing patient waiting or staff burden.

Prospective datasets should include patient identifiers, previous attendance history, procedure and specialty information, actual chair utilization, intervention delivery, cancellation timing, wait-list replacement, and operational costs. These variables would allow the framework to move from expected scheduled minutes at risk toward more direct estimates of avoidable capacity loss and cost-effectiveness.

Further research should also investigate provider-specific recalibration, hierarchical models, temporal drift monitoring, and periodic model updating. Equity analyses should assess whether prioritization changes access or intervention receipt across patient groups. Finally, the duration component could be extended to incorporate procedure-specific resource requirements, staffing constraints, clinical urgency, or different values assigned to unused appointment capacity.

## 5. Conclusions

This study demonstrates that the retrospective prioritization yield of dental no-show prediction depends not only on identifying appointments with the highest probability of non-attendance, but also on considering the amount of scheduled capacity associated with each appointment. Although the primary model showed moderate discrimination, combining Platt-calibrated no-show probability with scheduled appointment duration consistently identified a greater proportion of scheduled no-show minutes than probability-only or duration-only prioritization under the same intervention budget.

At the prespecified 10% intervention budget, duration-weighted prioritization captured 23.04% of scheduled minutes associated with no-shows, compared with 15.88% for probability-only targeting and 18.49% for duration-only targeting. This operational advantage was retained across intervention budgets, rolling-origin temporal evaluations, removal of exact duplicate records, exclusion of selected demographic predictors, and evaluation among previously unseen dentist identifiers.

The proposed scheduled-minutes-at-risk framework provides a simple and interpretable candidate ranking strategy for prioritizing appointments under hypothetical administrative targeting constraints. Its retrospective advantage remained evident under additional calibration-policy sensitivity analysis and dentist-cluster bootstrap resampling. However, the analysis did not establish actual reductions in non-attendance, recovery of clinical capacity, financial savings, or an economically optimal intervention budget. Independent external validation and prospective implementation studies are required to determine whether duration-weighted targeting improves attendance, capacity utilization, patient access, and cost-effectiveness in routine dental practice.

## Figures and Tables

**Table 1:** Dataset characteristics, data-integrity audit, and chronological partitioning.

Characteristic	Value
Total appointments	196,018
Study period	1 January–31 December 2019
Dentist identifiers	105
No-shows	83,663 (42.68%)
Total scheduled minutes	7,054,970
Scheduled minutes associated with no-shows	3,049,845 (43.23%)
Appointment duration, median	30 min
Appointment duration, range	5–300 min
Missing nationality	54,623 (27.87%)
Exact duplicate rows, excluding first occurrence	39,405 (20.10%)
Development set	121,966
Calibration set	42,626
Temporal test set	31,426
No-shows in temporal test set	13,772 (43.82%)
No-show-associated minutes in temporal test set	504,815

Note: Values are presented as *n*, *n* (%), or as otherwise indicated. Exact duplicate rows were defined across all 13 published variables and exclude the first occurrence of each identical record.

**Table 2:** Predictive performance and probability calibration in the independent temporal test set.

Model	Calibration	ROC-AUC	AP	Brier Score	Log Loss	Calibration Intercept	Calibration Slope	ECE
Logistic regression	Raw	0.6886	0.6087	0.2428	0.6848	0.673	0.951	0.1470
Logistic regression	Platt	0.6886	0.6087	0.2238	0.6382	0.301	1.089	0.0568
Logistic regression	Isotonic	0.6877	0.5992	0.2232	0.6379	0.245	1.009	0.0524

Note: AP, average precision; ECE, expected calibration error; ROC-AUC, area under the receiver operating characteristic curve. ECE was calculated across 10 quantile-based probability groups. The Platt-calibrated logistic regression was designated as the primary model.

**Table 3:** Operational performance of the four prioritization strategies at the 10% intervention budget in the independent temporal test set.

Strategy	Appointments Targeted	No-Shows Captured	No-Show Recall, %	No-Show Minutes Captured	Scheduled-Minute Recall, % (95% CI)	Precision, %	Number Needed to Contact	Scheduled No-Show Minutes Captured per 100 Targeted Appointments
Random expected	3143	1377.4	10.00	50,487.9	10.00	43.82	2.28	1606.4
Probability only	3143	2162	15.70	80,145	15.88 (15.37–16.38)	68.79	1.45	2550.0
Duration only	3143	1391	10.10	93,365	18.49 (17.54–19.05)	44.26	2.26	2970.6
Probability × duration	3143	1869	13.57	116,290	23.04 (22.33–23.70)	59.47	1.68	3700.0

Note: Values in parentheses are percentile-based 95% bootstrap confidence intervals based on 500 appointment-level replicates. Random targeting represents the analytical expectation under random appointment selection; consequently, expected counts may be non-integer. Scheduled-minute recall denotes the proportion of all scheduled minutes associated with no-shows captured among targeted appointments.

**Table 4:** Rolling-origin temporal validation of predictive performance and operational utility at the 10% intervention budget from July to December 2019.

Test Month	Test Appointments, *n*	No-Show Prevalence, %	ROC-AUC	AP	Brier Score	Probability-Only Minute Recall, %	Duration-Only Minute Recall, %	Duration-Weighted Minute Recall, %
July	13,167	41.10	0.7342	0.6693	0.2018	19.77	17.00	24.03
August	13,694	44.22	0.7350	0.6707	0.2058	20.01	21.95	27.87
September	20,954	43.83	0.6936	0.6026	0.2202	14.46	18.53	23.14
October	21,672	41.43	0.6842	0.5764	0.2188	15.08	18.04	22.85
November	15,418	43.61	0.6805	0.5995	0.2232	15.63	18.32	22.76
December	16,008	44.03	0.6991	0.6226	0.2183	16.34	17.55	23.59
Mean	—	43.03	0.7044	0.6235	0.2147	16.88	18.56	24.04

Note: AP, average precision; ROC-AUC, area under the receiver operating characteristic curve. Each monthly model was developed using earlier appointments, calibrated using the immediately preceding month, and evaluated in the subsequent test month. Scheduled-minute recall represents the proportion of all scheduled minutes associated with no-shows that was captured among appointments selected for intervention. The reported operational results correspond to the prespecified 10% intervention budget. The mean row represents the unweighted arithmetic mean across the six monthly test periods.

**Table 5:** Summary of secondary-model, dentist-transportability, demographic-exclusion, and duplicate-removal analyses.

Analysis	Test *n*	ROC-AUC	AP	Brier Score	Probability-Only Minute Recall, %	Duration-Only Minute Recall, %	Duration-Weighted Minute Recall, %
Primary logistic regression	31,426	0.6886	0.6087	0.2238	15.88	18.49	23.04
CatBoost	31,426	0.6873	0.6009	0.2212	17.54	18.49	23.21
Logistic regression without selected demographic variables	31,426	0.6886	0.6086	0.2238	15.83	18.49	22.98
Held-out dentists	7338	0.6372	0.5186	0.2298	12.17	19.76	22.24
Exact duplicates removed	30,786	0.6893	0.6084	0.2232	15.97	18.49	23.01

Note: All operational results refer to the 10% intervention budget. AP, average precision. The held-out-dentist analysis included 21 dentist identifiers not represented during model development or calibration.

## Data Availability

The dental appointment dataset analyzed in this study is publicly available as Supplementary File S4 of the original publication by Alabdulkarim et al. [4]. The dataset is provided as the comma-separated file peerj-cs-08-1147-s001.csv. No new patient-level data were collected or generated. The analysis code, machine-readable result tables, model outputs, and generated figures are provided in the Supplementary Materials accompanying this article.

## Supplementary Materials

The following supporting information can be downloaded at: https://www.mdpi.com/article/10.3390/healthcare14213731/s1: Table S1: Complete operational performance across intervention budgets; Table S2: Subgroup predictive and operational performance; Table S3: Dentist-specific predictive and operational performance; Table S4: Original appointment-level bootstrap estimates and confidence intervals; Table S5: Complete machine-readable specification of the primary prediction and calibration models; Table S6A–D: Distribution of exact duplicate occurrences by chronological data partition, calendar month, dentist identifier, and appointment hour; Table S7: Calibration-policy sensitivity analysis using raw, Platt-calibrated, and isotonic-calibrated probabilities; Table S8: Dentist-cluster bootstrap uncertainty analysis; Table S9: Incremental scheduled-minute capture across targeting budgets; Supplementary File S1: Original Google Colab analysis notebook; Supplementary File S2: Original reproducibility package containing analysis outputs, predictions, and figures; Supplementary File S3: Additional reproducibility package containing the reviewer-requested analyses, machine-readable Tables S6–S9, and the corresponding Python analysis script; Supplementary File S4: Source_Dataset_peerj-cs-08-1147-s001.csv.
