# Supplementary material for: From No-Show Prediction to Operational Action: A Retrospective Secondary Analysis of Calibrated, Duration-Weighted Dental Appointment Prioritization
Source: Healthcare (Basel). 2026 Nov 3;14(21):3731. doi: 10.3390/healthcare14213731 (PMC13763047; doi:10.3390/healthcare14213731)
Supplement: Supplementary file 1 [file healthcare-14-03731-s001.zip › Supplementary_Tables_S1-S4_and_S6-S9.pdf]

Supplementary Materials

From No-Show Prediction to Operational Action: A Calibrated, Duration-Weighted Framework for Dental Appointment Management

Table S1

Complete operational performance of random, probability-only, duration-only, and duration-weighted prioritization across intervention budgets ranging from 5% to 30%.

Budget, %	Strategy	Appointments targeted	No-shows captured	No-show recall, %	No-show minutes captured	Scheduled-minute recall, %	Precision, %	NNC	Minutes per 100 contacts
5	Random expected	1,572	688.9	5.00	25,252.0	5.00	43.82	2.28	1,606.4
5	Probability only	1,572	1,135	8.24	41,305	8.18	72.20	1.39	2,627.5
5	Duration only	1,572	689	5.00	51,245	10.15	43.83	2.28	3,259.9
5	Probability x duration	1,572	950	6.90	64,795	12.84	60.43	1.65	4,121.8
10	Random expected	3,143	1,377.4	10.00	50,487.9	10.00	43.82	2.28	1,606.4
10	Probability only	3,143	2,162	15.70	80,145	15.88	68.79	1.45	2,550.0
10	Duration only	3,143	1,391	10.10	93,365	18.49	44.26	2.26	2,970.6
10	Probability x duration	3,143	1,869	13.57	116,290	23.04	59.47	1.68	3,700.0
15	Random expected	4,714	2,065.8	15.00	75,723.9	15.00	43.82	2.28	1,606.4
15	Probability only	4,714	3,123	22.68	118,600	23.49	66.25	1.51	2,515.9
15	Duration only	4,714	2,046	14.86	132,665	26.28	43.40	2.30	2,814.3
15	Probability x duration	4,714	2,767	20.09	160,260	31.75	58.70	1.70	3,399.7
20	Random expected	6,286	2,754.8	20.00	100,975.9	20.00	43.82	2.28	1,606.4
20	Probability only	6,286	4,029	29.26	153,720	30.45	64.09	1.56	2,445.4
20	Duration only	6,286	2,743	19.92	173,440	34.36	43.64	2.29	2,759.1
20	Probability x duration	6,286	3,667	26.63	197,630	39.15	58.34	1.71	3,144.0
30	Random expected	9,428	4,131.7	30.00	151,447.7	30.00	43.82	2.28	1,606.4
30	Probability only	9,428	5,861	42.56	223,920	44.36	62.17	1.61	2,375.1
30	Duration only	9,428	4,186	30.40	235,905	46.73	44.40	2.25	2,502.2
30	Probability x duration	9,428	5,360	38.92	260,935	51.69	56.85	1.76	2,767.7

Note: Random targeting represents the analytical expectation under random appointment selection. NNC, number needed to contact to identify one no-show. Scheduled-minute recall denotes the proportion of all scheduled minutes associated with no-shows captured among targeted appointments.

Table S2

Predictive and operational performance according to sex, age group, and nationality-data availability in the independent temporal test set.

Subgroup variable	Subgroup	n	No-show prevalence, %	ROC-AUC	AP	Brier score	Probability-only minute recall, %	Duration-weighted minute recall, %
Sex	Female	8,576	43.03	0.6835	0.5898	0.2247	15.57	23.22
Sex	Male	22,850	44.12	0.6904	0.6152	0.2235	15.86	22.91
Age group	0-17	8,075	43.17	0.6590	0.5773	0.2296	16.85	23.42
Age group	18-34	12,780	45.09	0.6867	0.6236	0.2264	16.00	22.27
Age group	35-49	6,663	45.90	0.6993	0.6261	0.2248	14.20	22.06
Age group	50-64	3,114	38.60	0.7263	0.6076	0.2039	17.52	24.77
Age group	65+	794	33.25	0.7267	0.5500	0.1935	20.30	23.71
Nationality data	Missing	9,991	42.28	0.6909	0.5964	0.2209	16.09	23.16
Nationality data	Recorded	21,435	44.54	0.6872	0.6140	0.2252	15.80	22.95

Note: AP, average precision; ROC-AUC, area under the receiver operating characteristic curve. Operational results refer to the prespecified 10% intervention budget. Subgroup analyses were exploratory.

Table S3

Dentist-specific predictive and operational performance among dentist identifiers with at least 100 temporal test-set appointments and both outcome classes.

Dentist ID	n	No-show prevalence, %	ROC-AUC	AP	Brier score	Probability-only minute recall, %	Duration-weighted minute recall, %
1	394	43.15	0.6526	0.5307	0.2282	10.61	14.53
10	468	48.93	0.6257	0.5877	0.2385	12.21	12.21
101	135	60.00	0.7526	0.8098	0.2133	13.41	16.46
1023	110	48.18	0.7143	0.6498	0.2179	18.07	18.67
104	279	41.94	0.5765	0.4799	0.2484	13.50	13.50
105	144	44.44	0.7791	0.7341	0.2048	19.25	19.25
106	144	29.86	0.6391	0.4967	0.1967	27.54	33.33
108	125	23.20	0.6054	0.2920	0.1832	10.17	8.47
109	280	55.71	0.7360	0.7419	0.2456	14.15	15.11
11	507	54.64	0.7534	0.7627	0.2039	13.61	21.85
110	169	39.05	0.6333	0.4928	0.2273	12.17	12.93
111	178	42.70	0.5717	0.4981	0.2450	11.84	11.84
13	275	40.36	0.7134	0.5673	0.2084	17.08	21.20
14	469	42.86	0.6748	0.5623	0.2436	12.87	13.86
17	340	40.88	0.6898	0.5857	0.2228	15.87	24.30
18	1,100	42.36	0.6914	0.6013	0.2326	14.56	26.86
19	711	46.84	0.7074	0.6089	0.2144	10.42	22.35
2	412	48.30	0.6845	0.6277	0.2177	17.84	25.35
20	263	35.74	0.7843	0.7126	0.1631	16.85	25.59
21	474	43.04	0.6457	0.5461	0.2244	12.23	22.94
23	397	48.87	0.6783	0.6392	0.2278	17.11	18.09
24	756	42.46	0.7262	0.6297	0.2212	19.19	24.92
25	326	59.20	0.7384	0.7798	0.2029	13.44	23.77
26	644	45.50	0.7343	0.6708	0.2103	15.83	15.18
27	1,686	41.04	0.6214	0.5287	0.2348	12.50	19.33
28	355	36.90	0.6503	0.4928	0.2203	14.88	19.07
29	223	36.32	0.6293	0.5293	0.2204	26.35	27.76
3	226	39.38	0.7162	0.6018	0.2140	18.02	16.22
30	292	50.00	0.7546	0.7461	0.2064	14.65	25.78
31	500	41.60	0.6185	0.5049	0.2351	13.26	19.14
32	670	46.27	0.6539	0.6049	0.2323	15.20	15.20
33	222	45.95	0.6604	0.5685	0.2260	12.68	13.66
34	731	45.55	0.7066	0.6537	0.2299	18.61	19.64
35	267	51.69	0.7829	0.7946	0.2115	19.16	21.04
36	396	33.33	0.7249	0.5078	0.1931	18.70	22.22
38	225	39.11	0.6929	0.5630	0.2063	18.89	20.34
4	901	49.94	0.6678	0.6438	0.2373	14.21	18.95
41	539	39.89	0.6690	0.5466	0.2249	31.23	43.57
42	960	34.06	0.5528	0.3936	0.2232	10.94	21.01
44	493	41.99	0.6757	0.6137	0.2233	17.52	23.22
46	205	50.24	0.6207	0.5730	0.2514	12.53	13.49
47	180	47.22	0.6970	0.6367	0.2393	10.75	21.24
48	253	38.34	0.5842	0.4774	0.2347	14.70	24.64
49	319	40.75	0.6234	0.4980	0.2288	13.51	20.15
51	476	35.71	0.6521	0.4675	0.2167	14.30	19.49
52	408	50.00	0.7136	0.7014	0.2208	16.47	22.83
53	557	50.45	0.6523	0.6383	0.2373	15.66	15.66
54	347	45.82	0.6826	0.6131	0.2274	14.72	15.64
55	323	49.23	0.6423	0.5803	0.2404	12.29	17.17
58	304	39.80	0.6171	0.5154	0.2376	15.47	22.01
60	285	49.47	0.6655	0.6610	0.2360	17.12	19.37
61	184	43.48	0.7266	0.6621	0.2166	19.69	25.19

Table S3 (continued)

Dentist-specific predictive and operational performance among dentist identifiers with at least 100 temporal test-set appointments and both outcome classes.

Dentist ID	n	No-show prevalence, %	ROC-AUC	AP	Brier score	Probability-only minute recall, %	Duration-weighted minute recall, %
62	286	48.95	0.7763	0.7422	0.2010	17.92	26.54
63	210	38.10	0.6889	0.5841	0.2216	22.72	24.90
64	219	36.53	0.6900	0.5441	0.2116	18.99	23.89
65	271	44.65	0.6529	0.5814	0.2281	11.82	17.60
66	324	47.53	0.7321	0.6991	0.2108	15.95	21.77
67	352	51.70	0.7032	0.6860	0.2223	15.99	22.42
68	107	37.38	0.7280	0.6059	0.2002	19.95	21.48
69	451	41.69	0.7074	0.6313	0.2143	17.02	23.40
7	611	41.24	0.5808	0.4811	0.2393	16.07	19.75
70	327	40.06	0.6604	0.5342	0.2227	15.09	20.94
71	378	48.15	0.7135	0.6549	0.2153	14.33	15.33
72	214	30.37	0.6071	0.4052	0.2061	19.30	22.11
73	229	43.67	0.6306	0.5500	0.2322	15.37	11.71
74	315	46.98	0.6795	0.6272	0.2184	17.27	23.19
75	153	42.48	0.6747	0.5223	0.2421	11.43	17.14
77	185	46.49	0.7037	0.6104	0.2308	13.23	13.92
8	225	44.89	0.7660	0.6458	0.2075	15.02	28.83
80	322	49.07	0.5970	0.5528	0.2487	11.49	11.28
82	151	39.07	0.6967	0.5785	0.2360	13.64	27.27
83	232	35.78	0.6194	0.5067	0.2190	15.66	15.66
84	519	43.74	0.6803	0.6197	0.2279	13.94	22.25
85	252	42.46	0.6752	0.5887	0.2225	19.83	22.37
86	319	47.02	0.6233	0.5660	0.2401	14.29	16.07
88	308	37.01	0.6189	0.4806	0.2275	13.79	13.79
9	311	51.45	0.6810	0.6458	0.2216	14.50	13.02
91	147	45.58	0.7409	0.6643	0.2004	11.46	18.75
92	160	44.38	0.7487	0.7208	0.2089	19.72	19.72
93	354	45.20	0.7378	0.6410	0.2214	15.40	23.95
94	233	41.20	0.6293	0.5150	0.2263	14.94	26.05
95	318	53.46	0.6885	0.6947	0.2465	14.71	14.71
96	144	52.08	0.7766	0.8017	0.1988	20.13	20.13
98	390	46.41	0.6789	0.6178	0.2200	15.12	17.16
99	163	28.22	0.7187	0.4970	0.1861	22.89	24.22

Note: AP, average precision; ROC-AUC, area under the receiver operating characteristic curve. Operational results refer to the prespecified 10% intervention budget. Dentist identifiers are anonymized labels from the public dataset.

Table S4

Bootstrap estimates and percentile-based 95% confidence intervals based on 500 appointment-level bootstrap replicates.

Metric	Bootstrap mean	95% CI lower	95% CI upper	Replicates
ROC-AUC	0.6885	0.6827	0.6941	500
Average precision	0.6086	0.6001	0.6169	500
Brier score	0.2238	0.2220	0.2255	500
Probability-only scheduled-minute recall at 10%	15.89	15.37	16.38	500
Duration-only scheduled-minute recall at 10%	18.29	17.54	19.05	500
Duration-weighted scheduled-minute recall at 10%	23.02	22.33	23.70	500
Difference: duration-weighted minus probability-only scheduled-minute recall	7.13	6.47	7.79	500
Difference: duration-weighted minus duration-only scheduled-minute recall	4.72	4.00	5.39	500
Probability-only no-show recall at 10%	15.72	15.36	16.05	500
Duration-only no-show recall at 10%	9.98	9.57	10.37	500
Duration-weighted no-show recall at 10%	13.55	13.18	13.90	500

Note: Percentage-based metrics and contrasts are expressed in percentage points. The bootstrap mean is reported separately from the observed point estimate in the main analysis.

Supplementary Tables S6–S9: Additional Reviewer-Requested Analyses

Post hoc analyses using the original public dental appointment dataset and the primary model's saved independent temporal-test predictions.

These supplements describe retrospective appointment ranking and uncertainty, not delivered interventions, recovered chair time, financial benefits, or external validation. Tables S1–S4 and machine-readable Table S5 are retained as originally provided.

Table S6A

Exact duplicate occurrences by chronological data partition

Period	Records, n	Additional identical records, n	Duplicate occurrences, %
Development (Jan–Aug)	121,966	27,211	22.31
Calibration (Sep–Oct)	42,626	11,554	27.11
Test (Nov–Dec)	31,426	640	2.04
Total	196,018	39,405	20.10

Note: An "exact duplicate occurrence" denotes a row beyond the first identical row across all 13 original published variables, based on first-occurrence retention in the source CSV. Counts are descriptive and do not establish administrative recording errors.

Table S6B

Exact duplicate occurrences by appointment month, calendar year 2019

Month	Records, n	Duplicate occurrences, n	Duplicate occurrences, %
January	13,846	18	0.13
February	12,864	11	0.09
March	18,931	5,063	26.74
April	20,074	5,601	27.90
May	15,469	4,255	27.51
June	13,921	3,959	28.44
July	13,167	4,628	35.15
August	13,694	3,676	26.84
September	20,954	5,666	27.04
October	21,672	5,888	27.17
November	15,418	630	4.09
December	16,008	10	0.06
Total	196,018	39,405	20.10

Note: The marked temporal imbalance suggests a possible influence on effective observation weighting during model development and calibration; it does not establish the cause of duplication.

Table S6C

Exact duplicate occurrences by anonymized dentist identifier

Dentist ID	Records, n	Duplicate occurrences, n	Duplicate occurrences, %
18	8,184	3,144	38.42
27	11,358	3,084	27.15
19	6,514	2,647	40.64
10	6,122	2,564	41.88
7	5,232	2,077	39.70
2	3,535	1,424	40.28
98	3,420	1,398	40.88
11	3,654	1,385	37.90
28	3,477	1,383	39.78
14	3,340	1,275	38.17
26	4,597	1,157	25.17
32	5,144	1,098	21.35
35	2,778	1,080	38.88
23	2,805	1,077	38.40
25	2,597	988	38.04
30	2,355	936	39.75
20	2,377	923	38.83
24	4,379	874	19.96
34	4,045	863	21.33
17	2,041	804	39.39
13	2,073	784	37.82
8	2,018	783	38.80
31	3,123	689	22.06
3	1,837	686	37.34
29	1,640	643	39.21
9	2,480	636	25.65
21	2,900	632	21.79
33	1,620	623	38.46
36	2,575	618	24.00
101	1,296	554	42.75
38	1,614	507	31.41
1023	1,076	425	39.50
93	2,547	423	16.61
6	703	278	39.54
40	563	233	41.39
1008	544	232	42.65
102	461	190	41.21
109	456	99	21.71
104	831	52	6.26
97	206	47	22.82
108	171	30	17.54
42	4,656	7	0.15
4	3,897	5	0.13
41	2,824	4	0.14
54	1,837	4	0.22
70	1,663	3	0.18
71	1,937	3	0.15
72	1,158	3	0.26
88	1,850	3	0.16
95	2,037	3	0.15
49	1,662	2	0.12
53	2,723	2	0.07
62	1,439	2	0.14
75	827	2	0.24
1	1,813	1	0.06
103	261	1	0.38
105	351	1	0.28
39	6	1	16.67
44	2,763	1	0.04
51	2,379	1	0.04

Dentist ID	Records, n	Duplicate occurrences, n	Duplicate occurrences, %
60	1,739	1	0.06
64	1,023	1	0.10
65	1,356	1	0.07
66	1,715	1	0.06
68	622	1	0.16
74	1,737	1	0.06
80	1,823	1	0.05
83	1,126	1	0.09
86	1,690	1	0.06
92	950	1	0.11
94	1,177	1	0.08
100	225	0	0.00
106	232	0	0.00
107	139	0	0.00
110	169	0	0.00
111	178	0	0.00
112	82	0	0.00
37	235	0	0.00
43	1	0	0.00
45	48	0	0.00
46	1,012	0	0.00
47	861	0	0.00
48	1,446	0	0.00
52	2,086	0	0.00
55	1,618	0	0.00
56	22	0	0.00
57	158	0	0.00
58	1,741	0	0.00
61	912	0	0.00
63	608	0	0.00
67	1,763	0	0.00
69	2,338	0	0.00
73	1,175	0	0.00
76	433	0	0.00
77	755	0	0.00
78	464	0	0.00
79	752	0	0.00
81	62	0	0.00
82	659	0	0.00
84	2,633	0	0.00
85	1,208	0	0.00
90	62	0	0.00
91	826	0	0.00
96	759	0	0.00
99	627	0	0.00

Note: Dentist identifiers are anonymized labels in the source dataset. Table sorted by descending number of duplicate occurrences. The aggregate includes 105 dentist identifiers; 71 had at least one occurrence beyond the first identical record. The five largest duplicate counts sum to 13,516 (34.30% of all duplicate occurrences).

Table S6D

Exact duplicate occurrences by scheduled appointment hour

Appointment hour	Records, n	Duplicate occurrences, n	Duplicate occurrences, %
00:00–00:59	2,683	747	27.84
01:00–01:59	929	315	33.91
02:00–02:59	25	6	24.00
09:00–09:59	130	40	30.77
10:00–10:59	4,873	778	15.97
11:00–11:59	5,601	997	17.80
12:00–12:59	6,361	1,159	18.22
13:00–13:59	6,320	1,144	18.10
14:00–14:59	11,020	2,165	19.65
15:00–15:59	8,938	1,525	17.06
16:00–16:59	17,870	3,497	19.57
17:00–17:59	28,803	5,830	20.24
18:00–18:59	30,831	6,157	19.97
19:00–19:59	29,161	5,759	19.75
20:00–20:59	23,939	4,885	20.41
21:00–21:59	11,403	2,385	20.92
22:00–22:59	3,568	1,002	28.08
23:00–23:59	3,563	1,014	28.46
Total	196,018	39,405	20.10

Note: Appointment hours are those recorded in the original dataset; no chair-level timeline or actual appointment duration is available. For 17:00–20:59, 22,631 duplicate occurrences (57.43% of all duplicates) and 57.51% of all appointments were observed.

Table S7

Calibration-policy sensitivity, independent November–December 2019 test set

Panel A. Discrimination and probabilistic performance

Calibration	Test n	Observed no-show, %	Mean predicted, %	ROC-AUC	AP	Brier score
Raw	31,426	43.82	29.13	0.6886	0.6087	0.2428
Platt	31,426	43.82	38.14	0.6886	0.6087	0.2238
Isotonic	31,426	43.82	38.58	0.6877	0.5992	0.2232

Panel B. Prioritization at a 10% targeting budget

Calibration	Selected appointments, n	SMAR no-show minutes, n	SMAR minute recall, %	Probability-only minute recall, %	Overlap with Platt-selected, %
Raw	3,143	115,230	22.83	15.88	90.23
Platt	3,143	116,290	23.04	15.88	100.00
Isotonic	3,143	116,710	23.12	15.66	94.02

Note: Raw, Platt, and isotonic estimates are taken from the original saved predictions; calibration models were fitted using the September–October calibration period, and policies were evaluated exclusively in the November–December temporal test period. Overlap is the proportion of appointments in the Platt-selected set that were also selected by the specified method. The apparent stability of ranking does not establish adequate absolute-risk calibration or actual clinical benefits.

Table S8

Dentist-cluster bootstrap uncertainty, independent test set

Metric	Bootstrap mean	95% CI lower	95% CI upper	Replicates
Duration-weighted minute recall, %	23.01	21.67	24.33	1200
Probability-only minute recall, %	15.90	14.71	17.09	1200
Difference: SMAR – probability, pp	7.11	5.89	8.35	1200
ROC-AUC	0.6887	0.6759	0.7004	1200

Note: 1,200 post hoc bootstrap replicates, sampling with replacement from 96 unique dentist clusters in the independent November–December test set (seed 2026). Every sampled cluster contributes all its appointments; a cluster may be sampled repeatedly. Target selection was recomputed at a 10% budget in each resample. Intervals are percentile based. "pp" means percentage points. This method accounts for within-dentist grouping only; it is not a patient-level bootstrap and does not include uncertainty from refitting the model or calibration.

Table S9

Marginal scheduled-minute capture across intervention budgets

Budget, %	Selected, n	Captured no-show minutes, n	Minute recall, %	New contacts, n	Additional minutes, n	Minutes / extra contact
5	1,572	64,795	12.84	1,572	64,795	41.22
10	3,143	116,290	23.04	1,571	51,495	32.78
15	4,714	160,260	31.75	1,571	43,970	27.99
20	6,286	197,630	39.15	1,572	37,370	23.77
30	9,428	260,935	51.69	3,142	63,305	20.15

Note: The first row is the cumulative yield over the initial 5% selection. Each later row represents the incremental yield relative to the preceding budget. Values reflect retrospective identification of scheduled minutes associated with observed non-attendance, not intervention effectiveness, economic return, or recovered chair time.

Illustrative decision expression (not empirically estimated): $NB(b) = q \times v \times M(b) - c \times k(b)$, where $M(b)$ is retrospectively captured scheduled no-show minutes, $k(b)$ is targeted appointments, q is an unobserved recoverable fraction, v is assumed value per usable minute, and c is an assumed contact cost. No actual clinical or economic optimum can be identified from this dataset.

Computational reproducibility and interpretation

The original Supplementary File S1 notebook and its output CSVs are preserved without modification. A standalone Python script reproduces Tables S6A–S9 from the source public CSV and the primary independent temporal-test predictions. The source paper's publicly available CSV is required but intentionally not redistributed in this package. The additional analyses are post hoc and conditional on the original saved model predictions.

No independent external validation or prospective patient-contact intervention was performed. Neither scheduled-minute recall nor the illustrative net-benefit expression estimates realized recovery of clinical time.
